# Predicted structural mimicry of spike receptor-binding motifs from highly pathogenic human coronaviruses

**DOI:** 10.1016/j.csbj.2021.06.041

**Published:** 2021-07-02

**Authors:** Christopher A. Beaudoin, Arian R. Jamasb, Ali F. Alsulami, Liviu Copoiu, Andries J. van Tonder, Sharif Hala, Bridget P. Bannerman, Sherine E. Thomas, Sundeep Chaitanya Vedithi, Pedro H.M. Torres, Tom L. Blundell

**Affiliations:** a Department of Biochemistry, Sanger Building, University of Cambridge, Tennis Court Rd, Cambridge CB2 1GA, United Kingdom; b Department of Computer Science & Technology, University of Cambridge, JJ Thomson Ave, Cambridge CB3 0FD, United Kingdom; c Department of Veterinary Medicine, University of Cambridge, Madingley Rd, Cambridge CB3 0ES, United Kingdom; d King Abdullah International Medical Research Centre – Ministry of National Guard Health Affairs, Jeddah, Saudi Arabia; e King Saud bin Abdulaziz University for Health Sciences, Jeddah, Saudi Arabia; f Laboratório de Modelagem e Dinâmica Molecular, Instituto de Biofísica Carlos Chagas Filho, Universidade Federal do Rio de Janeiro, Rio de Janeiro, RJ, Brazil

**Keywords:** SARS-CoV-2, SARS-CoV, MERS-CoV, Coronavirus spike protein, COVID-19, Viral host mimicry, Infectious disease, Structural bioinformatics

## Abstract

Viruses often encode proteins that mimic host proteins in order to facilitate infection. Little work has been done to understand the potential mimicry of the SARS-CoV-2, SARS-CoV, and MERS-CoV spike proteins, particularly the receptor-binding motifs, which could be important in determining tropism and druggability of the virus. Peptide and epitope motifs have been detected on coronavirus spike proteins using sequence homology approaches; however, comparing the three-dimensional shape of the protein has been shown as more informative in predicting mimicry than sequence-based comparisons. Here, we use structural bioinformatics software to characterize potential mimicry of the three coronavirus spike protein receptor-binding motifs. We utilize sequence-independent alignment tools to compare structurally known protein models with the receptor-binding motifs and verify potential mimicked interactions with protein docking simulations. Both human and non-human proteins were returned for all three receptor-binding motifs. For example, all three were similar to several proteins containing EGF-like domains: some of which are endogenous to humans, such as thrombomodulin, and others exogenous, such as *Plasmodium falciparum* MSP-1. Similarity to human proteins may reveal which pathways the spike protein is co-opting, while analogous non-human proteins may indicate shared host interaction partners and overlapping antibody cross-reactivity. These findings can help guide experimental efforts to further understand potential interactions between human and coronavirus proteins.

## Highlights

• Potential coronavirus spike protein mimicry revealed by structural comparison.
• Human and non-human protein potential interactions with virus identified.
• Predicted structural mimicry corroborated by protein–protein docking.
• Epitope-based alignments may help guide vaccine efforts.

## Graphical abstract

**Figure:**
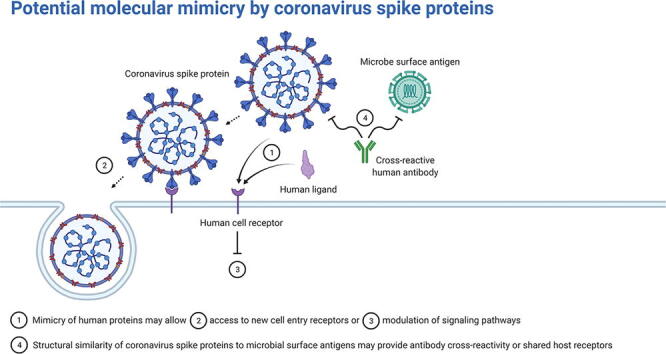

## 1 Introduction

Viruses have long been known to utilize molecular mimicry of host proteins to interrupt and exploit host biochemical pathways during infection [1], [2]. Alongside the need to employ host machinery for the viral replication cycle, the evolution of viral protein motifs that resemble host proteins can result in new virulence mechanisms, such as inducing inflammation and evading the immune system [3]. Coronaviruses, in particular, have been suspected to have acquired human protein mimics throughout the long record of human coronavirus infections [4], [5]. As further evidence, the highly pathogenic human coronaviruses, Severe Acute Respiratory Syndrome Coronavirus 2 (SARS-CoV-2), SARS-CoV, and the Middle Eastern Respiratory Syndrome Coronavirus (MERS-CoV), have been shown to encode numerous short linear motifs across their genomes that are homologous to human proteins [6]. Although coronavirus infections are typically localized to the lungs, resulting in respiratory infections, viral material has also been found in other organs, such as the kidney, brain, and heart, resulting in more life-threatening infections [7]. Furthermore, SARS-CoV-2 (the causative agent of the COVID-19 pandemic) infection has presented symptoms not previously seen in other coronavirus infections, such as conjunctival discharge from the eyes [8], [9]. Investigations into coronavirus host mimicry may shed light on viral tropism and infection severity [10].

The structure of the receptor-binding motif (RBM) on the spike glycoprotein is particularly important for determining the tropism of the virus [11]. Host receptors that contain motif(s) that complement the electrochemical and spatial configurations of the viral RBM will interact and, thus, initiate viral entry [12], [13]. Angiotensin converting enzyme II (ACE2) has been established as the primary cell entry receptor for SARS-CoV-2 and SARS-CoV and dipeptidyl peptidase IV (DPP4) as the primary cell entry receptor for MERS-CoV. However, several reports, some preliminary, have proposed additional coronavirus cell entry receptors, such as transferrin receptor protein 1, kidney injury molecule-1, kremen protein 1, and αv integrins for SARS-CoV-2 [14], [15], [16], [17], [18], [19], [20]. Additionally, coronavirus spike proteins have been proposed to interact with host factors to facilitate infection aside from their role in cell entry [21]. For instance, two studies found that the SARS-CoV-2 spike protein alone can interact with the blood brain barrier [22], [23]. The importance in receptor-binding and low glycosylation surrounding the coronavirus RBM residues make it an attractive target for inhibition by small-molecule drugs, therapeutic peptides, and neutralizing antibodies [24], [25], [26].

To date, there has been limited investigation into the structural similarity of highly pathogenic coronavirus RBMs [27]. Identifying structurally analogous human proteins may give insight into endogenous biochemical pathways that the virus is hijacking to facilitate infection or may help explain autoimmune disorders triggered by coronavirus infections [28], [29]. Detecting similar microbial proteins may reveal shared host receptors or antibody cross-immunity [30]. Short linear motifs on coronavirus spike RBMs have been shown to share high amino acid sequence identity with human proteins, which may indicate host mimicry [31], [32], [33], [34]. However, protein structure and fold similarity have been shown as more informative than amino acid sequence similarity in predicting molecular mimicry [35], [36]. Drayman *et al.* performed a structural similarity search using bacterial and viral motifs and experimentally validated the simian vacuolating virus 40 major capsid protein mimicry of Gas6 binding with TAM – Tyro3, Axl, and Mer – receptors, demonstrating that structural paralogs with low amino acid identity may still act as molecular mimics. Thus, to add to the understanding of host mimicry of highly pathogenic coronavirus RBMs, we used structural bioinformatics tools to model and map the extent to which the three-dimensional structures of the SARS-CoV-2, SARS-CoV, and MERS-CoV spike RBMs are potentially mimicking the interactions of experimentally-determined protein structures. We used structural alignment tools with distinct methodologies to perform a structural similarity screen between the RBMs and all known protein structures and, subsequently, tested potential RBM interactions with protein–protein docking simulations. Several cell signaling proteins, innate immune factors, snake and spider toxins, and microbial antigens are found to share structural features with the three RBMs. This information may help guide experimental efforts to elucidate spike RBM interactions, including that of vaccine design and cell entry receptor discovery.

## 2 Results and discussion

### 2.1 Receptor-binding motif structural similarities and characteristics

**Fig. 1:**
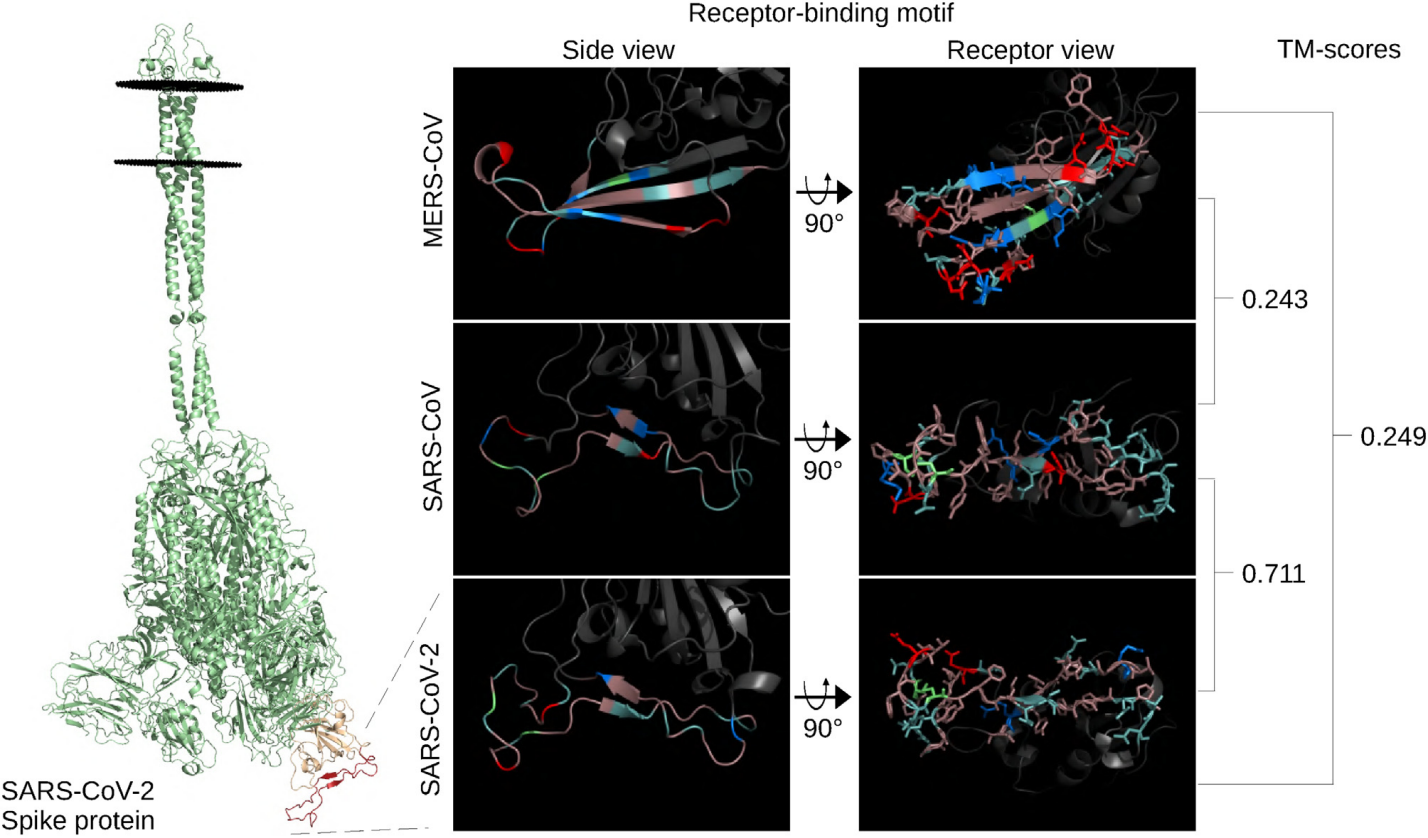
Spike receptor-binding motif comparison. The full-length SARS-CoV-2 spike protein (green), left, modelled using ProtCHOIR is shown with the receptor-binding domain (yellow) and receptor-binding motif (red) marked. The RBMs from the side view are shown, middle, with the amino acids labelled by color: red for acidic (D,E), blue for basic (H,R,K), light teal for polar non-charged (S,N,T,Q), dirty violet for hydrophobic (A,V,I,L,M,F,W,P,G,Y), and lime green for cysteine residues. RBMs from the host cell receptor side are shown, right, with amino acid stick configurations. (For interpretation of the references to color in this figure legend, the reader is referred to the web version of this article.)

Several models of the spike protein for each of the highly pathogenic coronaviruses have been experimentally determined; however, many of them are missing residues due to the difficulty in resolving the structure of flexible protein motifs [37]. To overcome this issue and obtain a representative three-dimensional model of each spike receptor-binding motif (RBM), we used ProtCHOIR, a recently developed pipeline to automate the modelling of homo-oligomers, to model each trimeric spike protein and, subsequently, manually selected the RBM residues for each coronavirus (Fig. 1). All generated models were structurally aligned to experimental models using TM-align to determine modelling precision. On a scale from 0 to 1, a TM-score of over 0.5 between two proteins implies that they have the same fold, while below 0.2 suggests a random alignment. Each RBM alignment with the corresponding experimental structure reported a TM-score over 0.95, reflecting high-quality modelling. Although receptor-binding of coronavirus spike proteins has been shown to be an elaborate process that involves interactions with glycans and multiple protein domains, we selected the most interactive region of the spike RBD with primary receptors (i.e. ACE2 for SARS-CoV and SARS-CoV-2; DPP4 for MERS-CoV) from experimental models as the receptor-binding motif (RBM) [38].

The structural similarity of the RBMs to one another was quantitatively assessed using TM-align before assessing their similarity to other known proteins. The SARS-CoV-2 and SARS-CoV RBMs were very similar with a TM-score of 0.71, while the TM-scores of MERS-CoV with the other two were both less than 0.25 (Fig. 1). This level of divergence is also reflected at the amino acid sequence level for the RBD of SARS-CoV-2 and SARS-CoV at 64.6% sequence identity and MERS-CoV with SARS-CoV-2 and SARS-CoV at 19% and 21.6%, respectively.

As seen in Fig. 1, the SARS-CoV-2 RBM is comprised almost exclusively of hydrophobic and polar non-charged amino acids, with the exception of one acidic glutamate and one basic lysine. SARS-CoV is similar to SARS-CoV-2 in that it is composed mostly of hydrophobic and polar non-charged residues with some exceptions as single amino acid differences, such as an acidic aspartate in the middle of the SARS-CoV RBM. The MERS-CoV RBM consists of more acidic and basic amino acids and contains fewer polar non-charged residues. Of note, the SARS-CoV-2 and SARS-CoV RBMs have 7 and 8 aromatic residues, respectively, exposed on the receptor-binding surface of the RBM. The recent discovery of the N501Y and E484K mutants add a potentially functional aromatic and basic residue, respectively, in the SARS-CoV-2 RBM – both of which have been proposed to increase binding to ACE2 [39]. Modelling of the mutants yielded very small structural changes in the SARS-CoV-2 RBM – TM-scores of the mutant RBMs aligned to the reference structure were above 0.9.

In terms of global architecture, the SARS-CoV-2 and SARS-CoV RBMs contain two anti-parallel beta-strands connecting three loops, although the SARS-CoV-2 RBM has two short beta-strands leading to a cystine disulfide loop (Fig. 1). Both SARS-CoV and SARS-CoV-2 contain a similar cystine disulfide bond helping shape one end of the respective RBMs. The MERS-CoV RBM consists of three beta-strands connecting four loops. Because loop flexibility may affect overall structure, we submitted each RBD to the CABS-flex 2.0 web server and found that the cystine disulfide loop of both the SARS-CoV-2 and SARS-CoV RBMs displayed high flexibility (>9 RMSF) - otherwise, the RBM residues on all three RBMs were predicted to exhibit low RMSF (less than6.5) (Supplementary Fig. 1). The flexibility predictions from CABS-Flex 2.0 were supported by separate studies on coronavirus RBMs [40], [41]. The high flexibility of the cystine loops in the SARS-related RBMs motivated the use of two additional models provided by CABS-Flex 2.0 for the structural similarity screen. The added models reported surprisingly low TM-scores compared to the references (0.42 and 0.65 for SARS-CoV-2 and 0.45 and 0.41 for SARS-CoV), revealing the high flexibility in these loops (Supplementary Fig. 1). Overall, SARS-CoV-2 and SARS-CoV were found to share higher structural homology with one another than in comparison with MERS-CoV.

### 2.2 Structural similarity screen

After RBM model generation, we performed a structural similarity screen for each RBM. Four sequence-independent 3D-structure alignment tools with different methodologies were used to quantify the structural similarity between the RBMs and known 3D protein structures in order to better understand shared structural features between the RBMs and potential mimics. Notably in this study, although spike may engage in interactions within human cells, we focused on protein structures that would be found in the extracellular matrix (excluding antibodies, due to their structural diversity) to gain more insight into potential cell entry receptors, immunopathies, and shared antigenicity with other microorganisms [42].

**Fig. 2:**
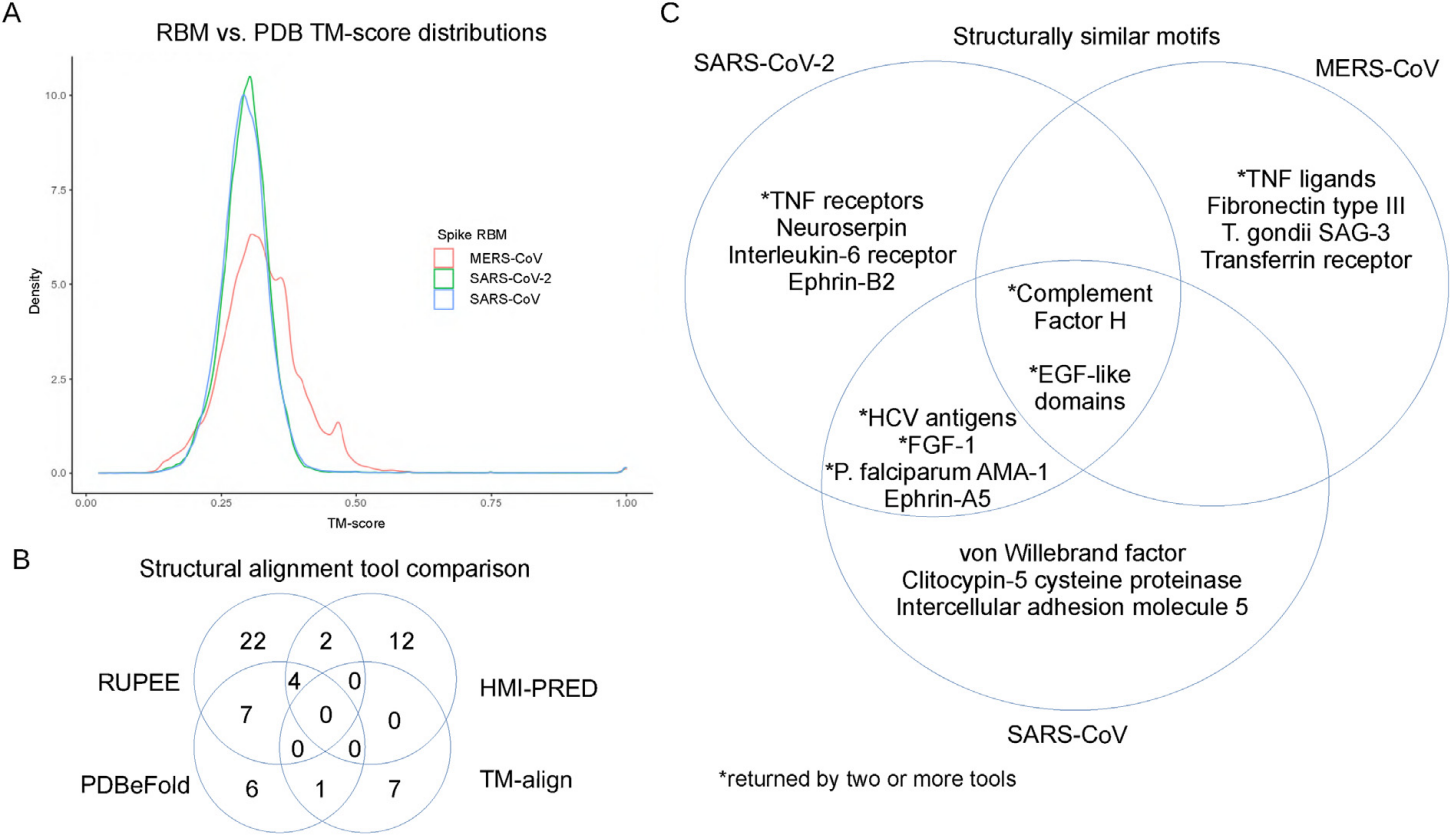
Summary of structural similarity screen. The TM-scores generated from the in-house TM-align screen are displayed as a density plot for each RBM (A). Number of shared proteins from the tools used in the structural similarity screen compared and contrasted (B). Structurally similar motifs, common between coronavirus receptor-binding motifs, compared and contrasted (C).

The PDBeFold, RUPEE, and HMI-PRED web servers were used, and TM-align was locally-installed and run pairwise against the downloaded PDB database clustered at 100% sequence identity. The TM-score distributions between SARS-CoV-2 and SARS-CoV were quite similar, while MERS-CoV was more similar to a greater number of proteins (Fig. 2A). The MERS-CoV RBM returned 3,954 structures with a TM-score of over 0.5 (~top 1% of TM-scores) out of 245,055 total RBM-chain alignments and an average TM-score of 0.33. The SARS-CoV-2 and SARS-CoV RBMs had lower average TM-scores, 0.298 and 0.297 respectively, and the top 1% corresponded roughly to the 0.4 TM-score line. Thus, structures with a TM-score of > 0.4 were selected for further analysis for the SARS-related viruses: 4,025 for SARS-CoV-2 and 3,561 for SARS-CoV. PDBeFold returned 621–806 and 1,163 structures for the SARS-related and MERS-CoV RBM models, respectively. The top 1,000 hits from each RUPEE run were recorded. HMI-PRED outputs ranged from 20 to 50 mimicked PDB templates per RBM. All alignments of interest were manually inspected to validate the potential for structural mimicry. Returned aligned proteins from each tool were linked to their corresponding PDB and UniProt codes. Shared UniProt codes between two or more tools were regarded as high-confidence hits. Biologically relevant structural alignments specific to each tool were also inspected and considered. Structural alignments that would not make sense biologically, such as when the RBM is facing the inside of the protein, were discarded, while alignments that were logical but found outside of protein–protein interfaces were included on a case-by-case basis. Returned structures not shown to be found in the extracellular matrix were removed. All tools returned their respective spike structures, confirming their validity.

A total of 62 UniProt codes, excluding 28 toxins, were considered as biologically relevant, which were comprised of 35, 19, 19, and 8 selected alignments from RUPEE, HMI-PRED, PDBeFold, and TM-align, respectively. When comparing tools (Fig. 2B), RUPEE and PDBeFold web servers shared 7 UniProt codes for at least one RBM, while TM-align shared 1 with PDBeFold and 0 with RUPEE. HMI-PRED shared 2 structures with RUPEE, 1 with PDBeFold, and 0 with TM-align. Little overlap was shown between most of the tools, which is consistent with structural similarity-based studies on HIV and human proteins [43]. The combined returned UniProt codes, excluding toxins, from all four tools totalled 39, 23, and 29 for the SARS-CoV-2, SARS-CoV, and MERS-CoV RBMs, respectively. The top alignments consisted of cytokines, chemokines, and growth factors and their receptors; structures containing EGF-like domains; complement activation proteins; cystine disulfide-rich toxins derived from snakes and spiders; and antigenic microbial proteins. A Venn diagram showing some shared hits between the three RBMs can be seen in Figure 2C, and a full listing of the hits, alignment values, and tools can be found in Table 1, Table 2. The SARS-CoV and SARS-CoV-2 RBMs shared more structural domains, while MERS-CoV returned more unique hits compared to the other two. Altogether these results indicate that proteins from completely different protein families may interact with coronavirus spike RBMs.

**Table 1:** Structural alignment values and data for endogenous hits.

Representative PDB								
MERS-CoV	SARS-CoV	SARS-CoV-2	Mimic UniProt Name	Alignment Length	Alignment Sequence Identity (%)	TM-score	RMSD	Tools
		1mox	Transforming growth factor alpha	25	4	0.25	3.53	R
1dx5	1zaq	1dqb	Thrombomodulin	21;20;25	4.8;5;4	0.31074;0.20501;0.2719	2.76;3.02;3.08	R,P and H (SARS-CoV-2)
5ky0		1toz	Neurogenic locus notch homolog protein 1	18;23	5.6;4.3	0.29958;0.26322	2.04;2.67	R,P
		5mwb	Neurogenic locus notch homolog protein 2	19	0	0.23683	2.81	R
1xka	2vwl	1fjs	Coagulation factor X	23;21	17.4;0	0.29888;0.24978	2.67;2.88	R,P
3th4	5ky2	1bf9	Coagulation factor VII	25;22	8;4.5	0.27601;0.25062	3.00;2.97	R,P
5f85		1edm	Coagulation factor IX	17;20	5.9;5	0.33731;0.25615	1.27;2.91	R,P
	3bt1	2fd6	Urokinase-type plasminogen activator	23	8.7	0.25522	3.13	R
		5wb7	Epiregulin	19;25	5.3;4	0.30636;0.23605	2.39;3.52	R
		5wb8	Epigen	22	13.6	0.34781	2.67	R
	1emn	1emn	Fibrillin	26;25;27	3.8;8;11.1	0.24425;0.26079	2.67;3.40;3.59	R
4hsv	1rhp	1nap	Platelet factor 4 (Platelet basic protein)	26;23;25	3.8;4.3;8	0.33673;0.19566;0.28516	2.67;3.77;2.97	R, H and P (SARS-CoV-2)
	2r3z	1o7z	Small inducible cytokine B10	27;28	0;7.1	0.28217;0.30758	3.38;2.9	R
	3oj2	3cu1	Fibroblast growth factor 1	37;21	5.4;14.3	0.29384;0.27446	3.54;2.74	H,P
	4c2b		von Willebrand factor	31	3.2	0.27715	3.53	H
	1b3e	1b3e	Serotransferrin	54;50	7.4;10	0.42052;0.41965	3.86;3.83	T
	4l0p	4m4r	Ephrin-A5	39;37	10.3;2.7	0.27482;0.31208	4.36;3.68	H
		2wo2	Ephrin-B2	34	8.8	0.3341	3.08	H
	1b53	1b50	C-C motif chemokine 3	26;22	0;0	0.24893;0.21513	3.13;2.97	R
		2h62	Bone morphogenetic protein 2	32	3.1	0.28716	3.69	H
	4xfu	4r6u	Interleukin-18	31;46	0;2.2	0.33910;0.34459	2.88;4.37	R
1qnk			C-X-C motif chemokine 2	27	7.4	0.2786	3.53	P
1je4			C-C motif chemokine 4	24	8.3	0.32333	3.12	P
3qb4			Growth/differentiation factor 5	39	17.9	0.35895	3.78	H
5mw5			Jagged-2	38	15.8	0.54617	2.56	T
4xbm			Delta-like protein 1	37	5.4	0.51041	2.32	T
5mvx			Delta-like protein 4	38	5.3	0.50604	2.47	T
		5fuc	Interleukin-6 receptor subunit alpha	36	0	0.34661	2.87	R
		1pvh	Interleukin-6 receptor beta chain	34	5.9	0.32838	3.51	R
4nqc			TCR beta chain	28	10.7	0.40641	2.42	H
5t5w			Interferon lambda receptor 1	27	7.4	0.37707	2.65	H
		2hey	Tumor necrosis factor receptor superfamily member 4	32	6.2	0.31459	3.33	R
		1ncf	Tumor necrosis factor receptor superfamily member 1A	24	4.2	0.29911	2.94	R
		2aw2	Tumor necrosis factor receptor superfamily member 14	25	12	0.23837	3.42	R
		1oqe	Tumor necrosis factor receptor superfamily member 13C	23	4.3	0.25858	3.29	R,P
3v56			Tumor necrosis factor ligand superfamily member 13B	40	12.5	0.39446	3.31	H,R
4en0			Tumor necrosis factor ligand superfamily member 14	37	16.2	0.44547	2.86	H,R
1hfh	5o32	1hfi	Complement factor H	33;35;28	9.1;2.9;3.6	0.3487;0.31744;0.27510	3.39;3.82;3.57	R,P (no SARS-CoV),H (SARS-CoV)
3oed			Complement receptor type 2 (CR2)	31	6.5	0.3175	3.23	H,R,P
6f1c			Complement C1s	23	8.7	0.38981	2.02	H
1x5y			Fibronectin type-III domain of mouse myosin-binding protein C	28	14.3	0.42255	2.45	R
2cum			Fibronectin type III domain of human Tenascin-X	29	3.4	0.40251	2.8	R
		3f5n	Neuroserpin	41	12.2	0.36905	3.58	H
	3h6s		Clitocypin-5 cysteine proteinase	34	2.9	0.32861	3.45	H
		2yhf	C-type lectin domain family 5 member A	30	3.3	0.30137	3.42	H
	3bn3		Intercellular adhesion molecule 5	39	3.34	0.36204	3.34	H
	3sq9	3u8m	Acetylcholine-binding protein	38;38	13.2;10.5	0.35462;0.38651	3.69;3.14	R

**Table 2:** Structural alignment values and data for exogenous hits.

Representative PDB									
MERS-CoV	SARS-CoV	SARS-CoV-2	Mimic UniProt Name	Species	Alignment Length	Alignment Sequence Identity (%)	TM-score	RMSD	Tools
		1n1i	Merozoite surface protein 1	Plasmodium knowlesi	22	4.5	0.25874	3.65	R
	2mgp	2mgp	Merozoite surface protein 1	Plasmodium yoelii	25;24	4;4.2	0.25287;0.27536	2.98;3.33	R
1cej		1ob1	Merozoite surface protein 1	Plasmodium falciparum	31;23	6.5;0	0.30361;0.24401	3.29;3.09	R
		1b9w	Merozoite surface protein 1	Plasmodium cynomolgi	24	4.2	0.26388	3.48	R
		2npr	Merozoite surface protein 1	Plasmodium vivax	24	4.2	0.24929	3.62	R
	1hn6	2j5l	Apical membrane antigen 1	Plasmodium falciparum	40;39	0;7.7	0.25857;0.26931	4.62;4.43	R,P
	1hky	1hky	Micronemal protein MIC4, related	Eimeria tenella (Coccidian parasite)	31;33	9.7;3	0.25771;0.31278	3.53;3.19	P
4yiz	4yiz	4yiz	Rhoptry neck protein 2, putative	Eimeria tenella (Coccidian parasite)	18;24;21	11.1;4.2;0	0.22386;0.28855;0.21409	2.79;3.29;3.36	P
2j4w			Apical membrane antigen 1	Plasmodium vivax	26	7.7	0.29687	2.84	R,P
2bbx			Thrombospondin-related anonymous protein	Plasmodium falciparum	25	4.8;13.5	0.30614	2.83	P
3sri			Rhoptry neck protein 2	Plasmodium falciparum (isolate 3D7)	17	5.9	0.28299	2.39	P
5wa2			Surface antigen 3	Toxoplasma gondii	36	8.3	0.50524	3.02	T
	4xvj	4g6a	E2 envelope glycoprotein	Hepatitis C virus	10;12	10;2.9	0.45252;0.37438	1.73;3.82	T,P
3kas			Transferrin receptor protein 1 / glycoprotein polyprotein GP complex	Machupo virus	38	10.5	0.51761	2.52	T
5f7l			Adhesin binding fucosylated histo-blood group antigen	Helicobacter pylori	37	2.7	0.53448	2.77	T
		2a2v	Kappa-theraphotoxin-Cg1a 1 (Jingzhaotoxin-XI)	Chilobrachys guangxiensis	18	0	0.23611	2.46	R
	2kni	2kni	Psalmotoxin-1	Psalmopoeus cambridgei	21;22	14.3;13.6	0.25651;0.25962	2.89;2.79	R,P
		1oma	Omega-agatoxin-Aa4b	Agelenopsis aperta	25	4	0.24137	3.07	R
		1g1p	Delta-conotoxin EVIA	Conus ermineus	17	5.9	0.22626	3.01	R
	1qdp	1qdp	Delta-hexatoxin-Ar1a (robustoxin)	Atrax robustus	26;23	7.7;0	0.25966;0.22613	3.76;3.7	R
		1la4	Kappa-theraphotoxin-Scg1a	Stromatopelma calceatum griseipes	22	13.6	0.22418	3.76	R
		2jtb	Hainantoxin-III 1	Haplopelma hainanum	18	5.6	0.22565	2.57	R
		1i26	Toxin Ptu1	Peirates turpis	21	9.5	0.25106	3.07	R
		1eit	Mu-agatoxin-Aa1a	Agelenopsis aperta	21	0	0.26268	2.73	R
		2mpq	Mu-theraphotoxin-Hd1a	Cyriopagopus doriae	18	5.6	0.27363	2.73	R
	1abt	2qc1	Alpha-bungarotoxin	Bungarus multicinctus	31;28	12.9;10.7	0.27217;0.33380	3.83;3.54	R
	4lft	4lft	Alpha-elapitoxin-Dpp2a	Dendroaspis polylepis polylepis	26	0	0.25472;0.32330	3.38;2.63	R
		2jqp	Weak toxin 1	Bungarus candidus	26;25	7.7;4	0.30834	3.36	R
		2nbt	Kappa-bungarotoxin	Bungarus multicinctus	28	3.6	0.27782	3.74	R
		2ctx	Long neurotoxin 3	Naja naja	28	3.6	0.32545	3.61	R
	1lxh		Long neurotoxin 1	Naja kaouthia	23	0	0.27252	2.85	R
	1txa		Long neurotoxin 2	Ophiophagus hannah	28	3.6	0.29226	3.28	R
	1c6w		Maurocalcin	Scorpio palmatus	18	0	0.27771	2.6	R
4om5			Cytotoxin 4	Naja atra	31	9.7	0.40943	2.71	R
4om4			Cytotoxin 2	Naja atra	29	10.3	0.40317	2.31	R
1onj			Cobrotoxin-b	Naja atra	30	10	0.38853	2.56	R
1era			Erabutoxin b	Laticauda semifasciata	34	17.6	0.3819	3.14	R
5ebx			Erabutoxin a	Laticauda semifasciata	36	16.7	0.38112	3.49	R
2mj4			Short neurotoxin 1	Naja oxiana	35	17.1	0.36641	3.33	R
1cod			Cobrotoxin homolog	Naja atra	34	11.8	0.35821	3.29	R
3hh7			Haditoxin	Ophiophagus hannah	34	8.8	0.37387	3.42	R
1jgk			Candoxin	Bungarus candidus	33	6.1	0.37726	3.47	R
1je9			Cobrotoxin-c	Naja kaouthia	31	16.1	0.34998	3.02	R

### 2.3 Analysis of predicted structural mimicry

Further examination of the structural alignments and their relevance to biological activity was performed to elucidate potential mechanisms of molecular mimicry by the SARS-CoV-2, SARS-CoV, and MERS-CoV spike RBMs. The UniProt and STRING databases were used to link the predicted mimics with potential interaction partners, and the PDB provided template structures to determine whether the alignments were found in ligand-binding regions. Selected high-confidence potential interactions were further evaluated using protein–protein docking with ClusPro PIPER in order to better understand electrochemical, in addition to structural, complementarity considering the low amino acid sequence identity. The docked models were then analyzed with the FoldX AnalyseComplex program to determine the complex interaction energy. Docking of the natural ligand to the receptor was performed to obtain a control interaction energy. The energy of the original PDB protein complex was also predicted as an experimental control. The exploration of these interactions with structural alignment visualization and protein–protein docking may help explain their potential roles in infection.

The potential mimics were split into two categories: endogenous vs. exogenous, or human vs. non-human, to more effectively describe the results in the context of infection. Mimicry of endogenous proteins may reveal which human pathways, specifically, the viral RBM is hijacking; structurally similar exogenous proteins may exhibit shared interference of human interaction pathways or antigenicity with the coronavirus RBMs. Endogenous hits, both discovered by single and multiple structural alignment tools, are summarized in Table 1 and exogenous hits in Table 2.

### 2.4 Endogenous

Several proteins containing EGF-like domains were found to be similar to all three RBMs. EGF-like domains are evolutionarily conserved domains that share homology to the epidermal growth factor and have been shown to function primarily in tissue organization and repair [44], [45]. Both the cystine disulfide loop and the central beta-strand sub-motif structures in the SARS-CoV-2 and SARS-CoV RBMs and the MERS-CoV beta-strands were found to mimic EGF-like domains.

The EGF-like domain of the urokinase-type plasminogen activator (uPa) in complex with its receptor, urokinase plasminogen activator receptor (uPAR), (PDB: 2fd6) was found to be similar to both SARS-CoV-2 and SARS-CoV RBMs using RUPEE. Interestingly, the uPa/uPAR system has been implicated in SARS-CoV-2 pathogenesis with uPAR as an early predictor of severe respiratory failure [46], [47]. Although the RBMs protrude into the receptor in the structural alignments, the alignments suggest that the RBMs might bind to uPAR (Supplementary Fig. 2A).

The neurogenic locus notch homolog protein 1 (NOTCH1) EGF-like domain was returned for the SARS-CoV-2 RBM central beta-strands and MERS-CoV RBM by RUPEE and PDBeFold. NOTCH1 is involved in developmental, innate immunity, and inflammation signaling pathways, and natural ligands of the NOTCH1 EGF-like domains include jagged-1, jagged-2, delta-like 1 (DLL1), DLL3, and DLL4 [48]. Alignment of the SARS-CoV-2 and MERS-CoV RBMs with the EGF-like domain of NOTCH1 bound to DLL4 (PDB: 4xl1) shows potential for molecular mimicry, i.e. the coronavirus RBMs may bind to DLL4 (Supplementary Fig. 2B) [49]. The SARS-CoV-2 RBM was also found similar to NOTCH2 by RUPEE, but no PDB complex models were available for further inspection. No direct interactions with the NOTCH1 pathway have been revealed, but its inhibition has been proposed to help fight SARS-CoV-2 infection [50].

All three RBMs were found to potentially mimic the EGF-like domain of coagulation factor VIIa. Further inspection of the alignment in complex with tissue factor (PDB: 1dan) showed potential for mimicry (Supplementary Fig. 2C) [51]. Interestingly, tissue factor expression has been shown to be up-regulated in severe SARS-CoV-2 infections, although there are several plausible theories [52], [53]. The cystine disulfide loops of SARS-CoV and SARS-CoV-2 were found to resemble the EGF-like domains of coagulation factors X and IX and fibrillin, which are known to bind calcium [54], [55]. However, there is no evidence for calcium binding to the RBMs.

**Fig. 3:**
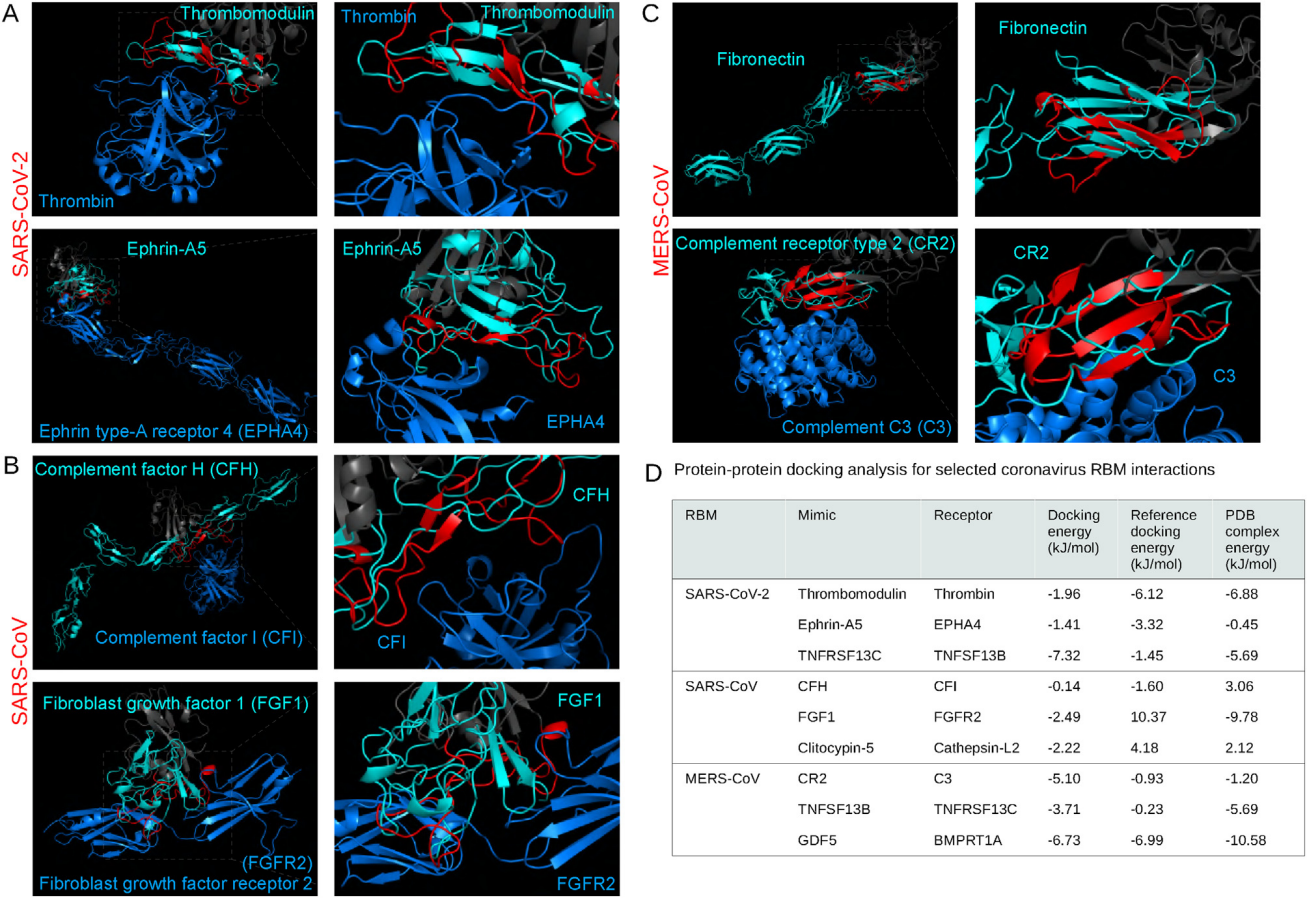
Analysis of endogenous structural alignments. Protein-protein docking was performed using ClusPro PIPER to test the potential interactions between the coronavirus RBMs and potential interaction partners. The following alignments are shown: between the SARS-CoV-2 RBM and thrombin (A, top) and ephrin type-A receptor 4 (A, bottom), the SARS-CoV RBM and complement factor I (B, top) and fibroblast growth factor receptor 2 (B, bottom), and the MERS-CoV RBM with complement C3 (C, bottom). The MERS-CoV RBM aligned to fibronectin type III domain (C, top). RBMs are labelled red, the remainder of the RBDs are dark gray, mimicked proteins are cyan, and potential interaction partners are marine blue. Interaction energy scores predicted using FoldX on docked and experimental complexes (D). (For interpretation of the references to color in this figure legend, the reader is referred to the web version of this article.)

All three RBMs were found to mimic the EGF-like domain of thrombomodulin, specifically in the region that binds thrombin (PDB: 1dx5), by RUPEE and PDBeFold, while the SARS-CoV-2 similarity was also detected by HMI-PRED [56]. Studies have shown that both thrombin and thrombomodulin blood concentrations are correlated with SARS-CoV-2 infection severity [57], [58]. The verification by three tools and relevance to the literature led us to explore the potential mimicking of thrombomodulin binding to thrombin by the SARS-CoV-2 RBM using protein–protein docking (Fig. 3A). Calculation of the interaction energies revealed that the reference docking and experimental controls showed similar affinities of −6.12 and −6.88 kJ/mol, and the SARS-CoV-2 RBM bound at a slightly lower affinity of −1.96 kJ/mol. The similarity to thrombomodulin might help explain the prothrombotic coagulopathy presented in SARS-CoV-2 infections [59].

The central beta-strands of the SARS-CoV-2 RBM were found to be structurally similar to the transforming growth factor alpha, epiregulin, and epigen EGF-like domains using RUPEE; however, alignment of the RBM with the proteins in complex with the epidermal growth factor receptor (EGFR) (PDBs: 1mox, 5wb7, 5wb8) showed that the RBM was just out of ligand-binding range (Supplementary Fig. 2E) [60], [61]. There is no evidence for interaction of the SARS-CoV-2 RBM with the extracellular domain of EGFR; however, the alignments were included for potential off-target effects related to the EGF-like domains.

Structural mimicry of chemokine and cytokine signaling has been reported for several viruses [1]. Viral proteins can mimic the chemokine, as in the case of HIV gp120 and CCL5, or they can mimic the receptor and bind directly to the cytokine (inhibiting its function), such as the vaccinia virus B15R protein that mimics the IL-1B receptor and binds to IL-1B [62], [63].

Several cell signaling ligands and receptors were found similar to the coronavirus RBMs. The SARS-CoV-2 and SARS-CoV RBMs were both found to mimic IL-8 like chemokines, fibroblast growth factor 1, C–C motif chemokines 3, interleukin-18, and ephrins; they also individually mimic BMP2 and von Willebrand factor, respectively. The MERS-CoV RBM structurally resembled C-X-C motif chemokines 2 and 4 and growth/differentiation factor 5. The alignments of the RBMs with IL-8 like chemokines, C–C motif chemokines 2 (CCL2), 3, and 4, and IL-18 in complex with their respective receptors shows only partial alignment with the ligand-binding regions (Supplementary Fig. 2F). Interestingly, however, expression levels of these cytokines have all been shown as correlating with SARS-CoV-2 infection, although other explanations have been proposed [64], [65], [66], [67]. For example, IL-33 release by damaged lower respiratory cells during SARS-CoV-2 has been demonstrated to trigger inflammation, increasing CCL2 and CCL3 expression [68].

Fibroblast growth factor 1 (FGF1) was shown to be similar to the SARS-CoV-2 and SARS-CoV RBMs using PDBeFold and HMI-PRED. A transcriptomic profiling revealed that FGF1 was upregulated in coronavirus infections [69]. Thus, to look more closely at potential interference, we docked the SARS-CoV RBM with the fibroblast growth factor receptor 2 (FGFR2) (PDB: 3OJ2), which was predicted by HMI-PRED (Fig. 3B) [70]. The RBM-FGFR2 docking analysis predicted a potentially favourable affinity of −2.49 kJ/mol, although not as high as the experimental complex (−9.78 kJ/mol) (Fig. 3D). The FGF1 signaling pathway may, thus, be modulated by the coronavirus spike RBMs.

HMI-PRED predicted that the SARS-CoV-2 RBM mimics ephrin-A5 and ephrin-B2 binding to the ephrin type 4a receptor (EPHA4), and SARS-CoV mimics ephrin-A5 binding to the ephrin receptor type 3a. EPHA4 is unique among known class A ephrin receptors in that it binds both ephrin a and b ligands [71], [72]. The structural similarity of two ligands for the same receptor for the SARS-CoV-2 RBM motivated further testing with protein–protein docking with EPHA4. Although there is no evidence for ephrin receptor involvement in coronavirus infections, other viral surface proteins have been shown to utilize ephrin receptors for cell entry, such as the rhesus r virus [73]. The docking revealed similar affinities between the SARS-CoV-2 RBM-EPHA4, ephrin-A5-EPHA4, and experimental (PDB: 4m4r) complexes: −1.41, −3.32, −0.45 kJ/mol, respectively (Fig. 3A).

The platelet glycoprotein Ib (GP-Ib) binding domain of von Willebrand factor (VWF) was found to be similar to SARS-CoV by HMI-PRED. VWF-GP-Ib interaction has been shown as critical in modulating thrombosis and inflammation [74]. Although there is no literature on VWF and SARS-CoV infection, blood concentration levels of VWF have been shown as correlated with SARS-CoV-2 infection severity, which may indicate potential pathway interference [75].

SARS-CoV-2 was predicted to mimic bone morphogenetic protein 2 binding to activin receptor type-2B and MERS-CoV to mimic growth/differentiation factor 5 (GDF5) binding to bone morphogenetic protein receptor type-1A (BMPRT1A) by HMI-PRED; however, no experimental evidence is available for either case. To explore the potential involvement of the MERS-CoV RBM in cell signaling, we docked the MERS-CoV RBM to the BMPRT1A in the GDF5-binding region (PDB: 3qb4) [76]. The docking of the RBM and GDF5 displayed similar affinities to BMPRT1A with −6.73 and −6.99 kJ/mol, respectively, while the experimental complex bound with −10.58 kJ/mol.

RUPEE detected structural resemblance between the SARS-CoV-2 RBM and IL-6 receptor alpha and beta chains, both of which show mimicry of the IL-6 binding sites (Supplementary Fig. 2G). IL-6 has been reported as an overexpressed cytokine in SARS-CoV-2 infections, which can lead to induction of a hyper-innate inflammatory response [77], [78]. Mimicry of the IL-6 receptors by the RBM could result in binding and, thus, interference of IL-6 related interactions. However, several alternative theories have been proposed to explain the increases in IL-6 during severe infection; for example, the SARS-CoV nucleocapsid protein has been shown to activate IL-6 expression [79]. HMI-PRED additionally predicted MERS-CoV RBM mimicry of the binding of the T cell receptor beta chain to the major histocompatibility complex class I-related gene protein and interferon lambda receptor 1 binding to the beta subunit of the interleukin-10 receptor, both of which could have implications in immunosurveillance and inflammatory pathways [80], [81].

Different tumor necrosis factor-related ligands and receptors were found to be structurally analogous to the MERS-CoV RBM and SARS-CoV-2 RBM, respectively. Tumor necrosis factor receptor superfamily (TNFRSF) 1A, 4, 13C, and 14 were returned for the cystine disulfide loop for the SARS-CoV-2 RBM by RUPEE, while the tumor necrosis factor ligand superfamily (TNFSF) 13B and 14 were found to resemble the MERS-CoV RBM by RUPEE and HMI-PRED. Similarity of the SARS-CoV-2 RBM to TNFRSF 13C was also found by PDBeFold. These signaling pathways normally promote B-cell and the T-cell survival and maturation [82], [83]. The structural similarity of this family of ligands and receptors to the SARS-CoV-2 and MERS-CoV RBMs led us to further inspect the interactions with protein–protein docking: mimicry of SARS-CoV-2 to TNFRSF 13C and MERS-CoV to TNFSF 13B. Thus, we simulated the binding of the SARS-CoV-2 RBM to TNFSF 13B and MERS-CoV to TNFRSF 13C (PDB: 3v56) [84]. Both cases revealed that the RBM is predicted to dock at a higher affinity than the natural ligand (Fig. 3D).

The complement system comprises a series of protein cascades that form an integral part of the innate immune response to viruses [85]. Viruses are generally susceptible to the complement system; however, viral proteins can utilize complement proteins through molecular mimicry in a variety of ways, such as using complement receptors for viral entry or evading detection by the immune system [86]. Infections from all three highly pathogenic coronaviruses have been reported to activate the complement system, enhancing pathogenicity, although the exact mechanisms remain unclear [87]. The spike protein of SARS-CoV-2 has been shown to localize near C4d and C5b-9 in lung vasculature, and mutations in several complement activation proteins, such as complement factors H, I, and III, have been found to correlate with infection severity [88], [89]. The structural similarity screen yielded three motifs from the complement system that potentially mimic RBMs: complement factor I (CFI) binding domain of CFH for all three RBMs and both the complement C3d binding domain of complement receptor 2 (CR2) and the complement C1r binding domain of complement C1s for the MERS-CoV RBM. Interestingly, CFH and the SARS-CoV-2 spike protein have been proposed to compete for heparan sulfate binding [90]. The SARS-CoV RBM, however, was predicted to be similar to CFH by RUPEE, PDBeFold, and HMI-PRED; thus, we docked the SARS-CoV RBM to CFI (PDB: 5o32) and found that the natural ligand was predicted to bind at a slightly higher affinity than the SARS-CoV RBM: −1.60 vs. −0.14 kJ/mol, respectively [91]. The C3d-binding domain of CR2 for the MERS-CoV RBM was also identified by RUPEE, PDBeFold, and HMI-PRED and was, thus, explored with docking of the MERS-CoV RBM to C3 (PDB: 3oed) (Fig. 3C) [92]. The MERS-CoV RBM was predicted to bind at a higher affinity than both the control docking and experimental complexes: −5.10 vs. −0.93 and −1.20 kJ/mol, respectively (Fig. 3D). Additionally, HMI-PRED found that the MERS-CoV RBM also mimics the complement C1r binding site of complement C1s. Additional experimental efforts are needed to validate the relationship between coronavirus spike proteins and the complement activation pathway.

Other endogenous hits included several unrelated proteins, such as protease inhibitors and serotransferrin. The MERS-CoV RBM resembled the fibronectin type III (FNIII) domains of mouse myosin-binding protein C and tenascin-X using RUPEE. Although myosin-binding protein C is intracellular, FNIII domains are found across the domains of life and function in diverse ways, from cell adhesion to cell signaling [93]. Drayman *et al.* found that the West Nile virus envelope glycoprotein E resembles the structural architecture of the FN10 domain of fibronectin, which is a natural ligand for integrin αvβ3. Thus, we checked and found that the MERS-CoV RBM shares structural properties with other FNIII domains, such as those from fibronectin and neural cell adhesion molecule 1 (PDBs: 2haz and 1fnf, respectively) (Fig. 3C) [94], [95]. The MERS-CoV RBM was also found to mimic part of the jagged-2, DLL1, and DLL4 proteins; however, the alignment was largely out of ligand-binding range when compared to jagged-1 in complex with NOTCH1 (PDB: 5uk5) – although the alignment may be relevant in other scenarios (Supplementary Fig. 2E) [96]. Protease inhibitors included neuroserpin for the SARS-CoV-2 RBM and clitocypin-5 cysteine protease for the SARS-CoV RBM. The alignment of the SARS-CoV with clitocypin-5 cysteine protease showed potential binding to cathepsin L2 (PDB: 3h6s) [97]. The role of cathepsins in coronavirus cell entry has been described as helping process the spike protein for viral and host membrane fusion [98]. To investigate the potential for additional interactions between coronavirus RBMs and cathepsins, we performed protein–protein docking. The binding of the SARS-CoV RBM to cathepsin L2 was predicted to be more favourable than the docking and experimental controls (Fig. 3D). Experimental evidence is required to validate this interaction, however. Both the SARS-related RBMs resembled motifs of serotransferrin using TM-align, and, interestingly, the transferrin receptor protein 1 has been proposed as a potential cell entry receptor for SARS-CoV-2 [17]. However, the alignments were generally out of ligand-binding range (PDB: 1suv) (Supplementary Fig. 2H); since no binding mode was apparent, it was not considered for docking [99]. HMI-PRED predicted that the SARS-CoV-2 RBM mimics the dimerization domain of C-type lectin domain family 5 and that the SARS-CoV RBM mimics intercellular adhesion molecule 5 binding to integrin alpha-L [100], [101]. Integrins have been proposed to bind to the SARS-CoV-2 spike protein, although that is due to a new RGD motif in the RBD – of note, the RGD motif is not included in the selected residues for this study’s SARS-CoV-2 RBM since it does not interact with ACE2 in experimental models [16]. Because integrin binding has not been hypothesized outside of the discussion of the SARS-CoV-2 RGD motif, docking was not pursued. Both the SARS-CoV-2 and SARS-CoV RBMs mimicked the nicotine-binding domain of the nicotinic acetylcholine receptor by RUPEE, which may have implications in the ‘nicotinic hypothesis’ [102].

### 2.5 Exogenous

We classified the exogenous hits by the pathogen type. There were motifs from apicomplexan parasites, viruses, one bacterial protein, and snake and spider toxins found to resemble the coronavirus RBMs.

**Fig. 4:**
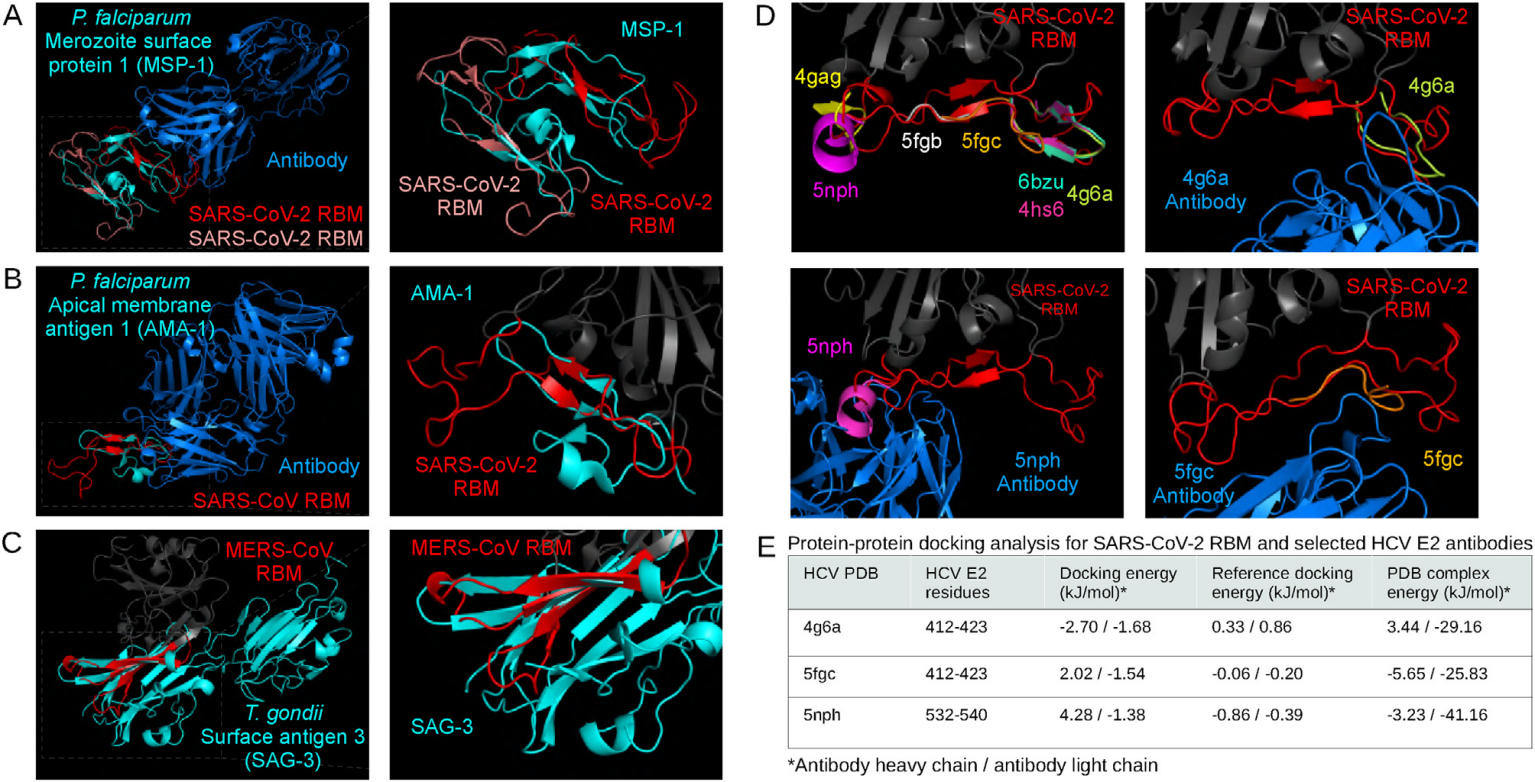
Analysis of exogenous structural alignments. *Plasmodium falciparum* merozoite surface protein 1 (A) and apical membrane antigen 1 (B) structurally aligned with the SARS-CoV-2 RBM and SARS-CoV RBM, respectively. The *Toxoplasma gondii* surface antigen 3 aligned with the MERS-CoV RBM (C). RBMs are labelled red, mimicked proteins are cyan, and potential interaction partners are marine blue (A, B, C). Hepatitis C virus epitopes structurally aligned to the SARS-CoV-2 RBM, and the respective antibody structures from PDBs 5fgc, 5nph, 4g6a docked to the RBM (D) using ClusPro PIPER “antibody” mode. Interaction energy scores predicted using FoldX on docked and experimental complexes (E). (For interpretation of the references to color in this figure legend, the reader is referred to the web version of this article.)

The EGF-like domains from merozoite surface protein 1 (MSP1) of several *Plasmodium* species were found to be structurally similar to all three RBMs using RUPEE. Compared to the other two, the SARS-CoV-2 RBM was found to be similar to the most *Plasmodium* species: *falciparum, yoelii, cynomolgi, knowlesi, vivax*. The SARS-CoV RBM returned *P. yoelii* MSP1 and the MERS-CoV RBM returned *P. falciparum* MSP1. A closer inspection at the *P.* f*alciparum* MSP1 alignments revealed that two EGF-like domains on the same PDB structure (1ob1) were found to resemble the SARS-CoV-2 RBM (Fig. 4A) [103]. The PDB structure is originally modelling the antibody-binding epitope of the EGF-like domain of MSP1; however, the antibody epitope is located on a loop just outside of the EGF-like domain. Thus, antibody-binding to the SARS-CoV-2 RBM could not be verified, but the presence of two EGF-like domains near an epitope may motivate experimental testing. The *P.* f*alciparum* apical membrane antigen 1 epitope (PDB: 2j5l) was also found to resemble the SARS-CoV-2 and SARS-CoV RBMs, although, as in the case of MSP1, both RBMs aligned to a region outside of the antibody-interacting residues (Fig. 4B) [104]. These EGF-like domains from *Plasmodium* parasites may provide structural epitope scaffolding for cross-reactivity against the coronavirus spike RBMs [105]. Recent studies have pointed to a potential protective effect of *P. falciparum* infections against SARS-CoV-2 infection, although direct experimental evidence is yet to be established [106], [107], [108], [109]. The MERS-CoV RBM was also found to resemble the rhoptry neck protein 2 and thrombospondin-related anonymous protein from *P. falciparum*. The surface antigen 3 of *Toxoplasma gondii* was found to be similar to the MERS-CoV RBM (Fig. 4C). Although there are no data on MERS-CoV and *T. gondii* co-infections, SARS-CoV-2 has been shown to have negative covariation with toxoplasmosis, which may indicate a protective effect from *T. gondii* [110].

The coronavirus RBMs were found to structurally mimic several motifs on the HIV and Influenza spike proteins; however, they were found either facing inwards or buried inside the mimicked protein and were, therefore, discarded. PDBeFold and TM-align indicated that the SARS-CoV-2 and SARS-CoV RBMs structurally mimic several hepatitis C virus (HCV) antibody epitopes. The SARS-CoV-2 and SARS-CoV RBMs were found to be similar to 10 and 6 PDB HCV E2 protein epitopes structures, respectively (Supplementary Table 2). The HCV E2 protein is implicated in host entry, which has been explored as an inhibitory target with neutralizing antibodies [111], [112]. A closer inspection of the mapping of the epitopes to the SARS-CoV-2 RBM show that they are distributed across the RBM (Fig. 4D). Some studies have suggested that HCV may be negatively correlated with SARS-CoV-2 infection [113], [114], [115]. Since several of the epitopes were aligned in ways that were accessible to antibodies in the original PBD, we selected three epitopes, one at each region of the RBM, and docked the respective antibody to the SARS-CoV-2 RBM using the ClusPro PIPER ‘antibody’ mode (Fig. 4D). As shown in Fig. 4E, the RBM-antibody docking results were compared to docking and experimental controls – the antibodies bound in a similar way to docking controls in all three cases, while the experimental complexes were predicted to bind more tightly. These structural similarities may take part in potential cross-reactivity between HCV and coronavirus infections. Of note, two recently proposed cell entry receptors for the SARS-CoV-2 spike protein, ASGR1 and APOA4, have been shown as potentially implicated in mediating HCV viral entry [116]. In an interesting case, the MERS-CoV RBM was found to structurally mimic both the Machupo virus glycoprotein polyprotein GP complex RBM (TM-score: 0.47) and its receptor, transferrin receptor protein 1 (0.52) (PDB: 3kas) using TM-align, although the transferrin receptor scores slightly higher (Supplementary Fig. 3A) [117].

Only one bacterial protein was selected in the structural similarity screen. The adhesin-binding fucosylated histo-blood group antigen of *Helicobacter pylori* was found to be similar to the MERS-CoV RBM by TM-align. The structure (PDB: 5f7l) shows binding of the bacterial protein to a nanobody; however, the RBM alignment is just outside of the nanobody binding site (Supplementary Fig. 3B) [118]. No studies have detailed any connections between MERS-CoV and *H. pylori*.

Motifs from snake, spider, and cone snail toxins were found to be similar to all three RBMs using PDBeFold and RUPEE. The SARS-CoV-2 and SARS-CoV RBMs shared similarity to four toxins, and the MERS-CoV RBM only returned unique proteins. The two SARS-related viruses mimicked three-finger bungarotoxins and inhibitor cystine-knot toxins, such as psalmotoxin-1, while MERS-CoV RBM resembled other three-finger toxins, like cytotoxin 4 (Supplementary Fig. 3C, D) [119], [120], [121], [122], [123]. In total, these toxins may bind to several receptors involved in nociception, e.g. ASIC1 and Nav1.7, which may be relevant to the taste and pain perception changes experienced during SARS-CoV-2 infection [124]. Importantly, and perhaps confoundingly, a recent study found no changes in depolarization for Nav1.7 and Cav2.2 upon exposure to the SARS-CoV-2 RBD [125]. Thus, further experimental work is necessary to validate these interactions.

## 3 Methods

### 3.1 Spike receptor-binding motif model generation and characterization

Amino acid sequences of the SARS-CoV-2 (NCBI code: NC_045512), SARS-CoV (NC_004718), and MERS-CoV (NC_038294) spike proteins were extracted as FASTA files from the NCBI Viral Genomes Resource [133]. Each amino acid sequence corresponds to one of three identical protomers of the full homo-oligomeric spike trimer. Due to the high number of available experimentally-resolved structures for each spike protein, representative models were generated using ProtCHOIR – a recently developed bioinformatic tool to automate 3D homology modelling of homo-oligomers [134]. ProtCHOIR builds homo-oligomeric assemblies by searching for homolog templates on a locally created homo-oligomeric protein database using PSI-BLAST, performing a series of structural analyses on the input protomer structure or sequence using Molprobity, PISA, and GESAMT (all three tools as part of the CCP4 Molecular Graphics package), and comparative homology modelling using MODELLER (version 9.24) with molecular dynamics-level optimization and refinement [135], [136], [137], [138], [139], [140]. Trimerization was detected for all three coronavirus spike proteins.

The residues for the receptor-binding domains (RBD) and RBMs of each spike model were manually selected based on experimental structures with primary receptors (residues defined in Supplementary Table 1) and made into sub-structures during manual inspection of full-length models on PyMOL (The PyMOL Molecular Graphics System, Version 2.0 Schrödinger, LLC). Global amino acid sequence alignment of RBDs was performed with EMBOSS Needle [141]. The full-length SARS-CoV-2 spike protein modelled with the lipid bilayer displayed in Fig. 1 was retrieved from the SARS-CoV-2 3D database [142]. No records exist of N-linked or O-linked glycosylation motifs near the three RBMs, which was supported by NetNGlyc 1.0 and NetOGlyc 4.0 predictions [143], [144]. To determine the flexibility of each residue in the RBDs, we used CABS-flex 2.0, a web server that offers fast simulations and resulting data of protein structure flexibility [145]. Default values were used and residue flexibility was reported as root mean squared flexibility (RMSF) [146].

### 3.2 Structure similarity screen

Several web servers and stand-alone tools have become available to perform pairwise or multiple sequence-independent protein structure alignments, such as DALI, FATCAT, iSARST, MADOKA, PDBeFold, TM-align, and RUPEE [147], [148], [149], [150], [151], [152], [153]. After testing each tool, the PDBeFold web server, RUPEE web server, and a locally-installed version of TM-align were selected due to the diversity of structural alignment methodologies, ease-of-use, data accessibility, and widespread-usage. A newly published web server for structural prediction of host-microbe interactions based on interface mimicry, HMI-PRED, was also included in the analysis.

Of note, mTM-align (the web server version of TM-align) was considered, but no non-spike proteins were shown – restricting the downstream analysis [154]. Thus, all 3D models in the PDB database clustered at 100% sequence identity were downloaded, and TM-align was run in a pairwise manner, using GNU parallel, between each RBM model and each chain of every downloaded PDB file (O. Tange (2018): GNU Parallel, March 2018, https://doi.org/10.5281/zenodo.1146014.). TM-align works by, first, combining secondary structure similarity alignments, defined by DSSP (Define Secondary Structure of Proteins), and TM-score-based structural alignments [155], [156]. A structure rotation matrix is applied to the alignments in order to maximize the TM-score, which was used to rank the alignments for each RBM.

The PDBeFold web server utilizes SSM, a graph-matching algorithm that superimposes *PROMOTIF*-defined secondary structures and, subsequently, maps backbone carbon atoms of, first, matched and, second, unmatched secondary structures [151], [157], [158]. The hits are ranked by their Q-Score, which is calculated to achieve a lower root mean squared deviation (RMSD) and an increased number of aligned residues. Since the highest percentage (%) of secondary structure matches for the SARS-CoV RBM was found at 67% (while SARS-CoV-2 and MERS-CoV returned hits with 100% secondary structure matches), we set the PDBeFold search parameters for 65% structural similarity at “highest precision” for each RBM.

The RUPEE web server performs structural similarity comparisons using a purely geometric approach: 1) a linear encoding of the protein structure is defined to identify separable regions of permissible torsion angles for DSSP secondary structure assignments; 2) the encoding is converted into a bag of features; 3) a protein structure indexing method is established using min-hashing and locality sensitive hashing; 4) the top 8,000 matches are sorted based on adjusted Jaccard similarity scores; and 5) if running in “Top-Aligned” mode (used in this analysis), the alignments are re-scored using TM-align [153]. RUPEE allows the specific comparison of a query protein to the CATH, SCOP, PDB, and ECOD databases [159], [160], [161], [162]. Additional settings are also offered, such as the “contains” (finding query protein inside database protein) and “contained in” options (small protein motif detection in query protein) – both of which were used in this analysis.

HMI-PRED combines TM-align with NACCESS to search through template host protein-protein complexes [163], [164]. The structural alignment model of the ligand and putative receptor are refined with RosettaDock to quantify electrochemical complementarity, which is not included in a strict structural alignment screen [165]. Alignments for both the RBM and RBD of each of the three coronaviruses were collected from HMI-PRED.

Since the flexibility of the cystine disulfide loop on the SARS-CoV-2 and SARS-CoV RBMs may affect the global structure of the RBMs and, thus, search outcome, we used two additional models provided by CABS-flex 2.0 for both RBMs, making a total of three conformations for each SARS-CoV-2 and SARS-CoV. Results of the different conformations provided were pooled together for both SARS-related RBMs. The top-scoring alignments from all four tools for each RBM were matched with their corresponding PDB and, subsequently, UniProt accession code [166]. The UniProt accession codes were then compared across tools to identify shared top hits.

### 3.3 Protein-protein docking and interaction energy prediction

A local installation of ClusPro PIPER (version 1.1.5) was used for protein-protein docking [167], [168], [169], [170]. Annotations informing potential protein-protein interactions were obtained from the PDB, STRING, and UniProt databases [171]. The ClusPro PIPER “antibody” docking mode was used to dock RBDs with the hepatitis C antibody PDB structures (PDBs: 4g6a, 5fgc, 5nph), and the “others” mode was used for all non-antibody docking. In order to minimize non-biologically relevant binding during the docking runs, residues outside of the RBM on the spike RBDs and outside of the ligand-binding region on the predicted interaction partners were masked. The docked models were minimized using CHARMM22. To gain better insight into the binding strength of the potential RBD-receptor complex interactions, we used the FoldX (version 4.0) AnalyseComplex program, which predicts the interaction energy by finding the difference in stability between the individual unfolded molecules and the overall complex [172]. The original PDB ligands were also docked to the receptor in order to obtain a “Reference docking energy” when compared with the predicted RBD-receptor energy. The “PDB complex energy” was obtained from the original PDB containing the ligand and receptor to understand binding resolution of the experimental complex.

### 3.4 Data analysis and visualization

**Fig. 5:**
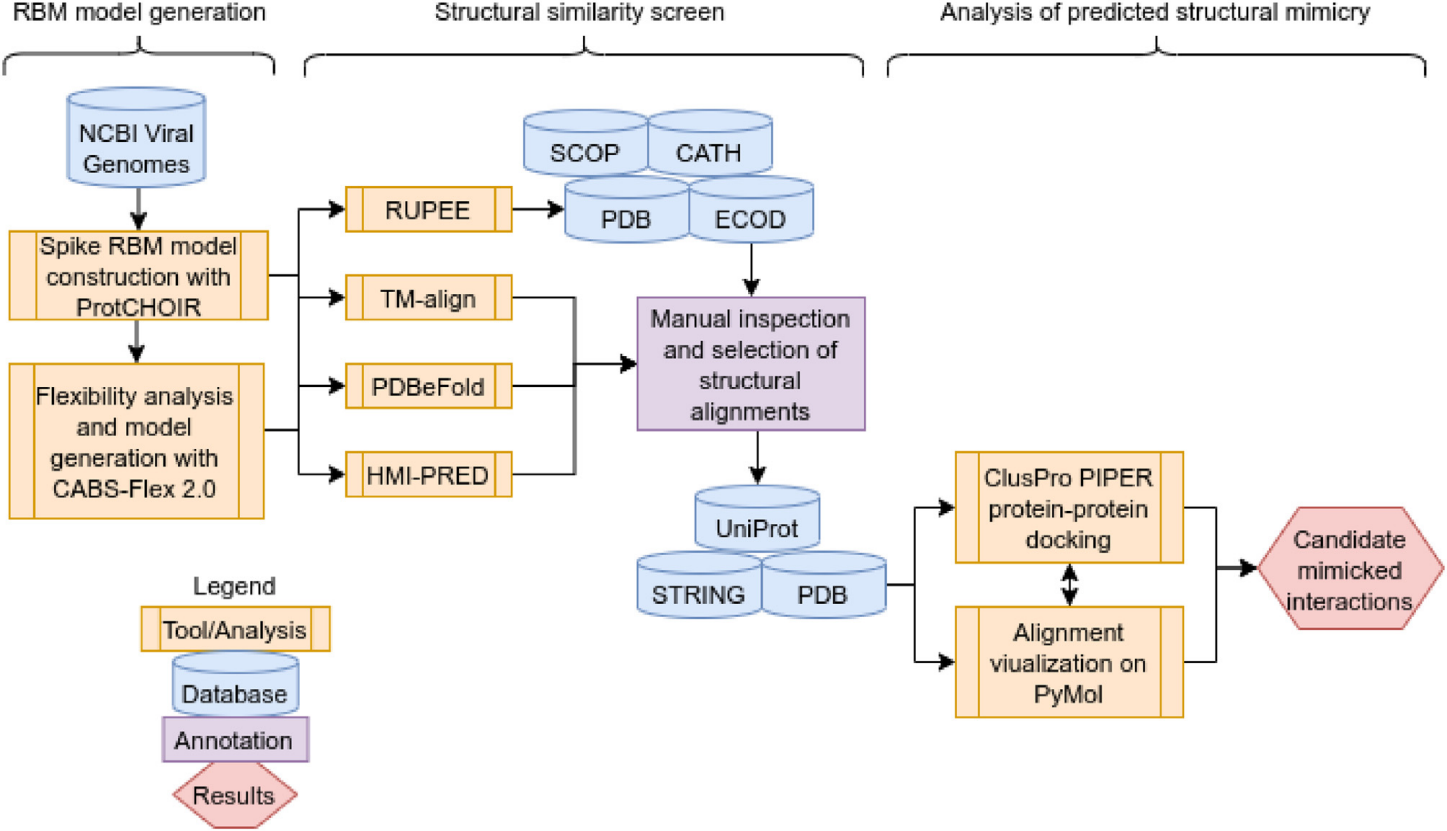
Pipeline flow. A flow chart of the analyses performed in this study.

A full representation of the pipeline and tools used can be found in Fig. 5. Data were analyzed and plots were generated using R version 3.6.3 (2020-02-29). Protein structural alignments were visualized with PyMOL (version 1.8.4.0) [173]. Pdb-tools was used to manipulate and organize PDB files [174]. The graphical abstract was adapted from the “SARS-CoV-2 Spike Protein Conformations” template on BioRender. Fig. 5 was created on draw.io. Raw data and alignment models are made available at https://github.com/tlb-lab.

## 4 Conclusions

This study involved the structural bioinformatics characterization of potential molecular mimicry by highly pathogenic coronavirus spike protein RBMs. Using protein homology modelling, we built representative models of the spike RBMs and tested structural changes in the SARS-CoV-2 RBM induced by recently recorded mutations, which had little effect on overall RBM structure. Comparison of the RBMs revealed that the SARS-CoV and SARS-CoV-2 RBMs share higher structural homology than with MERS-CoV, which was underlined by the number of common returned proteins in the structural similarity screen using four structural alignment tools. The flexibility of the cystine disulfide loop in the SARS-related RBMs was found to permit large global changes in RBM structure; however, since most of the predicted mimicry was mapped to the RBM central beta-strands, which are quite rigid, the models of different conformations did not return significantly different proteins from the structural alignment tools. The structural alignment screen highlighted the similarity of the RBMs to evolutionarily unrelated human and non-human proteins. Further validation of the alignments with protein–protein docking revealed that all tested coronavirus RBM-endogenous protein interactions were predicted to be energetically favourable, confirming that the structural similarity screen may be useful in identifying potential molecular mimics.

The predicted endogenous mimicry comprised of proteins in cell signaling, adhesion, and complement pathways. Potential mimicry of several microbial antigenic proteins and exogenous toxins was also discovered. The EGF-like domains of both endogenous and exogenous proteins structurally resemble all three RBMs. Predicted mimicked endogenous interactions include the EGF-like domain of thrombomodulin binding to thrombin, NOTCH1 binding to DLL4, and coagulation factor VIIa binding to tissue factor. Interference in these pathways may partially explain coagulopathies in coronavirus infections [126]. Exogenous EGF-like domains of MSP1 from different *Plasmodium* species, on the other hand, may provide a structural epitope scaffold for cross-reactivity between coronavirus and *Plasmodium* infections [106]. Epitope similarity was further explored among the several antibody-bound hepatitis C virus E2 protein motifs that were structurally analogous to the SARS-related RBMs. Structural similarity to antigenic proteins from other microbes may confer cross-immunity and, thus, also potentially guide vaccine design [127]. Cell signaling pathway proteins, such as TNF-related and ephrin ligands, were also found as potential mimics of the coronavirus RBMs, which may lead to use of alternative co-receptors for viral entry or modulation of signaling cascades. Complement factor H was returned for all three RBMs and has also been implicated in coronavirus infections [90]. The mimicry of complement proteins is widespread among viruses, and the spike RBM may have secondary roles interfering in these pathways [128]. Many snake and spider toxins were also found similar to the coronavirus RBMs, which implies the potential usage of receptors involved in pain, muscle contraction, cell adhesion, and coagulation pathways [129], [130], [131]. The prediction of evolutionarily unrelated, yet structurally similar, potential protein mimics reveals that previously unidentified pathways could be altered by the spike RBMs. The structural variation between coronavirus RBMs and their resulting molecular mimics can possibly be connected to differences in tropism, infection severity, and immune system reactivity between coronaviruses.

Although experimental verification of the predicted interactions is required to take these results further, the findings presented in this study provide insight into the potential molecular mimicry utilized by highly pathogenic coronavirus RBMs. The data can be used to support inhibitory drug, peptide, and antibody design efforts in order to prevent viral cell entry and virulence mechanisms related to coronavirus RBMs [132]. Additional work is needed to better understand how coronaviruses co-opt host machinery to enhance fitness.

## 5 Permission note

Permission has been granted to use all text, illustrations, charts, tables, photographs, or other material from previously published sources.

## CRediT authorship contribution statement

**Christopher A. Beaudoin:** Conceptualization, Methodology, Validation, Formal analysis, Investigation. **Arian R Jamasb:** Methodology, Formal analysis, Investigation. **Ali F. Alsulami:** Formal analysis, Investigation, Resources. **Liviu Copoiu:** Validation, Formal analysis, Investigation. **Andries J. van Tonder:** Software, Resources. **Sharif Hala:** Software, Resources. **Bridget P. Bannerman:** Software, Resources. **Sherine E. Thomas:** Software, Resources. **Sundeep Chaitanya Vedithi:** Software, Funding acquisition. **Pedro H.M. Torres:** Software, Validation, Supervision. **Tom L. Blundell:** Supervision, Project administration.

## Declaration of Competing Interest

The authors declare that they have no known competing financial interests or personal relationships that could have appeared to influence the work reported in this paper.

## References

[1] Alcami A. (2003). Viral mimicry of cytokines, chemokines and their receptors. Nat Rev Immunol.

[2] Lasso G., Honig B., Shapira S.D. (2021). A sweep of earth’s virome reveals host-guided viral protein structural mimicry and points to determinants of human disease. Cell Syst.

[3] Elde N.C., Malik H.S. (2009). The evolutionary conundrum of pathogen mimicry. Nat Rev Microbiol.

[4] Walls A.C., Xiong X., Park Y.-J., Tortorici M.A., Snijder J., Quispe J. (2019). Unexpected receptor functional mimicry elucidates activation of coronavirus fusion. Cell.

[5] Tim Chew F., Ong S.Y., Hew C.L. (2003). Severe acute respiratory syndrome coronavirus and viral mimicry. Lancet.

[6] Martínez Y.A., Guo X., Portales-Pérez D.P., Rivera G., Castañeda-Delgado J.E., García-Pérez C.A. (2021). The analysis on the human protein domain targets and host-like interacting motifs for the MERS-CoV and SARS-CoV/CoV-2 infers the molecular mimicry of coronavirus. PLoS ONE.

[7] Renu K., Prasanna P.L., Valsala G.A. (2020). Coronaviruses pathogenesis, comorbidities and multi-organ damage – A review. Life Sci.

[8] Cheong K.X. (2020). Systematic Review of Ocular Involvement of SARS-CoV-2 in Coronavirus Disease 2019. Curr Ophthalmol Rep.

[9] Zheng B, Yuan M, Ma Q, Wang S, Tan Y, Xu Y, et al. Landscape of SARS-CoV-2 spike protein-interacting cells in human tissues. Int Immunopharmacol 2021:107567. https://doi.org/https://doi.org/10.1016/j.intimp.2021.107567. 10.1016/j.intimp.2021.107567 PMC7945790 33756225

[10] Angileri F., Legare S., Marino Gammazza A., Conway de Macario E., JL Macario A., Cappello F. (2020). Molecular mimicry may explain multi-organ damage in COVID-19. Autoimmun Rev.

[11] Reguera J., Mudgal G., Santiago C., Casasnovas J.M. (2014). A structural view of coronavirus–receptor interactions. Virus Res.

[12] Tortorici M.A., Veesler D. (2019). Structural insights into coronavirus entry. Adv Virus Res.

[13] Li F., Goff S.P. (2015). Receptor recognition mechanisms of coronaviruses: a decade of structural studies. J Virol.

[14] T. Ichimura Y. Mori P. Aschauer K.M. Padmanabha Das R.F. Padera A. Weins et al. KIM-1/TIM-1 is a Receptor for SARS-CoV-2 in Lung and Kidney MedRxiv 2020:2020.09.16.20190694. 10.1101/2020.09.16.20190694.

[15] Yang C, Zhang Y, Chen H, Chen Y, Yang D, Shen Z, et al. Kidney injury molecule-1 is a potential receptor for SARS-CoV-2. BioRxiv 2020:2020.10.09.334052. https://doi.org/10.1101/2020.10.09.334052.

[16] Sigrist CJ, Bridge A, Le Mercier P. A potential role for integrins in host cell entry by SARS-CoV-2. Antiviral Res 2020;177. https://doi.org/10.1016/j.antiviral.2020.104759. 10.1016/j.antiviral.2020.104759 PMC7114098 32130973

[17] Tang X, Yang M, Duan Z, Liao Z, Liu L, Cheng R, et al. Transferrin receptor is another receptor for SARS-CoV-2 entry. BioRxiv 2020:2020.10.23.350348. https://doi.org/10.1101/2020.10.23.350348.

[18] Li W., Moore M.J., Vasilieva N., Sui J., Wong S.K., Berne M.A. (2003). Angiotensin-converting enzyme 2 is a functional receptor for the SARS coronavirus. Nature.

[19] Wang N., Shi X., Jiang L., Zhang S., Wang D., Tong P. (2013). Structure of MERS-CoV spike receptor-binding domain complexed with human receptor DPP4. Cell Res.

[20] Gu Y, Cao J, Zhang X, Gao H, Wang Y, Wang J, et al. Interaction network of SARS-CoV-2 with host receptome through spike protein. BioRxiv 2020:2020.09.09.287508. https://doi.org/10.1101/2020.09.09.287508.

[21] Patra T., Meyer K., Geerling L., Isbell T.S., Hoft D.F., Brien J. (2020). SARS-CoV-2 spike protein promotes IL-6 trans-signaling by activation of angiotensin II receptor signaling in epithelial cells. PLOS Pathog.

[22] Buzhdygan T.P., DeOre B.J., Baldwin-Leclair A., Bullock T.A., McGary H.M., Khan J.A. (2020). The SARS-CoV-2 spike protein alters barrier function in 2D static and 3D microfluidic in-vitro models of the human blood–brain barrier. Neurobiol Dis.

[23] Rhea E.M., Logsdon A.F., Hansen K.M., Williams L.M., Reed M.J., Baumann K.K. (2021). The S1 protein of SARS-CoV-2 crosses the blood–brain barrier in mice. Nat Neurosci.

[24] Ling R., Dai Y., Huang B., Huang W., Yu J., Lu X. (2020). In silico design of antiviral peptides targeting the spike protein of SARS-CoV-2. Peptides.

[25] Pandey P., Rane J.S., Chatterjee A., Kumar A., Khan R., Prakash A. (2020). Targeting SARS-CoV-2 spike protein of COVID-19 with naturally occurring phytochemicals: an in silico study for drug development. J Biomol Struct Dyn.

[26] Hussain A., Hasan A., Nejadi Babadaei M.M., Bloukh S.H., Chowdhury M.E.H., Sharifi M. (2020). Targeting SARS-CoV2 spike protein receptor binding domain by therapeutic antibodies. Biomed Pharmacother.

[27] Kanduc D., Shoenfeld Y. (2020). Molecular mimicry between SARS-CoV-2 spike glycoprotein and mammalian proteomes: implications for the vaccine. Immunol Res.

[28] Drayman N., Glick Y., Ben-nun-Shaul O., Zer H., Zlotnick A., Gerber D. (2013). Pathogens use structural mimicry of native host ligands as a mechanism for host receptor engagement. Cell Host Microbe.

[29] Angileri F, Légaré S, Marino Gammazza A, Conway de Macario E, Macario AJL, Cappello F. Is molecular mimicry the culprit in the autoimmune haemolytic anaemia affecting patients with COVID-19? Br J Haematol 2020;190:e92–3. https://doi.org/https://doi.org/10.1111/bjh.16883. 10.1111/bjh.16883 PMC7283741 32453861

[30] Huang A.T., Garcia-Carreras B., Hitchings M.D.T., Yang B., Katzelnick L.C., Rattigan S.M. (2020). A systematic review of antibody mediated immunity to coronaviruses: kinetics, correlates of protection, and association with severity. Nat Commun.

[31] An H, Park J. Molecular Mimicry Map (3M) of SARS-CoV-2: Prediction of potentially immunopathogenic SARS-CoV-2 epitopes via a novel immunoinformatic approach. BioRxiv 2020:2020.11.12.344424. https://doi.org/10.1101/2020.11.12.344424.

[32] Grifoni A., Sidney J., Zhang Y., Scheuermann R.H., Peters B., Sette A. (2020). A sequence homology and bioinformatic approach can predict candidate targets for immune responses to SARS-CoV-2. Cell Host Microbe.

[33] Lin L, Ting S, Yufei H, Wendong L, Yubo F, Jing Z. Epitope-based peptide vaccines predicted against novel coronavirus disease caused by SARS-CoV-2. Virus Res 2020;288. https://doi.org/10.1016/j.virusres.2020.198082. 10.1016/j.virusres.2020.198082 PMC7328648 32621841

[34] Lucchese G., Flöel A. (2020). Molecular mimicry between SARS-CoV-2 and respiratory pacemaker neurons. Autoimmun Rev.

[35] Westall F.C. (2006). Molecular mimicry or structural mimicry?. Mol Immunol.

[36] Krishna S.S., Grishin N.V. (2004). Structurally analogous proteins do exist!. Structure.

[37] Nwanochie E., Uversky V.N. (2019). Structure determination by single-particle cryo-electron microscopy: only the sky (and Intrinsic Disorder) is the Limit. Int J Mol Sci.

[38] Wang L., Xiang Y.e. (2020). Spike glycoprotein-mediated entry of SARS coronaviruses. Viruses.

[39] Nelson G, Buzko O, Spilman P, Niazi K, Rabizadeh S, Soon-Shiong P. Molecular dynamic simulation reveals E484K mutation enhances spike RBD-ACE2 affinity and the combination of E484K, K417N and N501Y mutations (501Y.V2 variant) induces conformational change greater than N501Y mutant alone, potentially resulting in an escap. BioRxiv 2021:2021.01.13.426558. https://doi.org/10.1101/2021.01.13.426558.

[40] Saputri D.S., Li S., van Eerden F.J., Rozewicki J., Xu Z., Ismanto H.S. (2020). Flexible, functional, and familiar: characteristics of SARS-CoV-2 spike protein evolution. Front Microbiol.

[41] Spinello A., Saltalamacchia A., Magistrato A. (2020). Is the rigidity of SARS-CoV-2 spike receptor-binding motif the hallmark for its enhanced infectivity? insights from all-atom simulations. J Phys Chem Lett.

[42] Versteeg G.A., van de Nes P.S., Bredenbeek P.J., Spaan W.J.M. (2007). The coronavirus spike protein induces endoplasmic reticulum stress and upregulation of intracellular chemokine mRNA concentrations. J Virol.

[43] Doolittle J.M., Gomez S.M. (2010). Structural similarity-based predictions of protein interactions between HIV-1 and Homo sapiens. Virol J.

[44] Engel J. (1989). EGF-like domains in extracellular matrix proteins: Localized signals for growth and differentiation?. FEBS Lett.

[45] Tombling B.J., Wang C.K., Craik D.J. (2020). EGF-like and other disulfide-rich microdomains as therapeutic scaffolds. Angew Chemie Int Ed.

[46] D’Alonzo D., De Fenza M., Pavone V. (2020). COVID-19 and pneumonia: a role for the uPA/uPAR system. Drug Discov Today.

[47] Rovina N., Akinosoglou K., Eugen-Olsen J., Hayek S., Reiser J., Giamarellos-Bourboulis E.J. (2020). Soluble urokinase plasminogen activator receptor (suPAR) as an early predictor of severe respiratory failure in patients with COVID-19 pneumonia. Crit Care.

[48] Shang Y., Smith S., Hu X. (2016). Role of Notch signaling in regulating innate immunity and inflammation in health and disease. Protein Cell.

[49] Luca VC, Jude KM, Pierce NW, Nachury M V, Fischer S, Garcia KC. Structural basis for Notch1 engagement of Delta-like 4. Science (80-) 2015;347:847 LP – 853. https://doi.org/10.1126/science.1261093. 10.1126/science.1261093 PMC4445638 25700513

[50] Rizzo P., Vieceli Dalla Sega F., Fortini F., Marracino L., Rapezzi C., Ferrari R. (2020). COVID-19 in the heart and the lungs: could we “Notch” the inflammatory storm?. Basic Res Cardiol.

[51] Banner D.W., D'Arcy A., Chène C., Winkler F.K., Guha A., Konigsberg W.H. (1996). The crystal structure of the complex of blood coagulation factor VIIa with soluble tissue factor. Nature.

[52] Eslamifar Z., Behzadifard M., Soleimani M., Behzadifard S. (2020). Coagulation abnormalities in SARS-CoV-2 infection: overexpression tissue factor. Thromb J.

[53] Bautista-Vargas M., Bonilla-Abadía F., Cañas C.A. (2020). Potential role for tissue factor in the pathogenesis of hypercoagulability associated with in COVID-19. J Thromb Thrombolysis.

[54] Handford P., Downing A.K., Rao Z., Hewett D.R., Sykes B.C., Kielty C.M. (1995). The calcium binding properties and molecular organization of epidermal growth factor-like domains in human fibrillin-1. J Biol Chem.

[55] Stenflo J., Stenberg Y., Muranyi A. (2000). Calcium-binding EGF-like modules in coagulation proteinases: function of the calcium ion in module interactions. Biochim Biophys Acta.

[56] Fuentes-Prior P., Iwanaga Y., Huber R., Pagila R., Rumennik G., Seto M. (2000). Structural basis for the anticoagulant activity of the thrombin–thrombomodulin complex. Nature.

[57] Ranucci M., Sitzia C., Baryshnikova E., Di Dedda U., Cardani R., Martelli F. (2020). Covid-19-associated coagulopathy: biomarkers of thrombin generation and fibrinolysis leading the outcome. J Clin Med.

[58] Goshua G., Pine A.B., Meizlish M.L., Chang C.-H., Zhang H., Bahel P. (2020). Endotheliopathy in COVID-19-associated coagulopathy: evidence from a single-centre, cross-sectional study. Lancet Haematol.

[59] Bongiovanni D., Klug M., Lazareva O., Weidlich S., Biasi M., Ursu S. (2021). SARS-CoV-2 infection is associated with a pro-thrombotic platelet phenotype. Cell Death Dis.

[60] Freed D.M., Bessman N.J., Kiyatkin A., Salazar-Cavazos E., Byrne P.O., Moore J.O. (2017). EGFR ligands differentially stabilize receptor dimers to specify signaling kinetics. Cell.

[61] Garrett T.P.J., McKern N.M., Lou M., Elleman T.C., Adams T.E., Lovrecz G.O. (2002). Crystal structure of a truncated epidermal growth factor receptor extracellular domain bound to transforming growth factor. Cell.

[62] Alcami A., Smith G.L. (1992). A soluble receptor for interleukin-1β encoded by vaccinia virus: a novel mechanism of virus modulation of the host response to infection. Cell.

[63] Ahuja S.K., Murphy P.M. (1999). Chemokines in Disease.

[64] Chen I.-Y., Chang S.C., Wu H.-Y., Yu T.-C., Wei W.-C., Lin S. (2010). Upregulation of the chemokine (C-C Motif) ligand 2 via a severe acute respiratory syndrome coronavirus spike-ACE2 signaling pathway. J Virol.

[65] Zaid Y., Puhm F., Allaeys I., Naya A., Oudghiri M., Khalki L. (2020). Platelets Can associate with SARS-CoV-2 RNA and are hyperactivated in COVID-19. Circ Res.

[66] Jamilloux Y., Henry T., Belot A., Viel S., Fauter M., El T. (2020). Should we suppress or stimulate immune responses for covid-19. Autoimmun Rev.

[67] Buszko M., Park J.-H., Verthelyi D., Sen R., Young H.A., Rosenberg A.S. (2020). The dynamic changes in cytokine responses in COVID-19: a snapshot of the current state of knowledge. Nat Immunol.

[68] Zizzo G., Cohen P.L. (2020). Imperfect storm: is interleukin-33 the Achilles heel of COVID-19?. Lancet Rheumatol.

[69] Alsamman A.M., Zayed H., Schindler M. (2020). The transcriptomic profiling of SARS-CoV-2 compared to SARS, MERS, EBOV, and H1N1. PLoS ONE.

[70] Beenken A., Eliseenkova A.V., Ibrahimi O.A., Olsen S.K., Mohammadi M. (2012). Plasticity in interactions of fibroblast growth factor 1 (FGF1) N terminus with FGF receptors underlies promiscuity of FGF1. J Biol Chem.

[71] Xu K, Tzvetkova-Robev D, Xu Y, Goldgur Y, Chan Y-P, Himanen JP, et al. Insights into Eph receptor tyrosine kinase activation from crystal structures of the EphA4 ectodomain and its complex with ephrin-A5. Proc Natl Acad Sci 2013;110:14634 LP – 14639. https://doi.org/10.1073/pnas.1311000110. 10.1073/pnas.1311000110 PMC3767517 23959867

[72] Bowden T.A., Aricescu A.R., Nettleship J.E., Siebold C., Rahman-Huq N., Owens R.J. (2009). Structural plasticity of eph receptor A4 facilitates cross-class ephrin signaling. Structure.

[73] Wang J., Zheng X., Peng Q., Zhang X., Qin Z. (2020). Eph receptors: the bridge linking host and virus. Cell Mol Life Sci.

[74] Denorme F., Vanhoorelbeke K., De Meyer S.F. (2019). von willebrand factor and platelet glycoprotein Ib: A thromboinflammatory axis in stroke. Front Immunol.

[75] Klok F.A., Kruip M.J.H.A., van der Meer N.J.M., Arbous M.S., Gommers D.A.M.P.J., Kant K.M. (2020). Incidence of thrombotic complications in critically ill ICU patients with COVID-19. Thromb Res.

[76] Klammert U., Mueller T.D., Hellmann T.V., Wuerzler K.K., Kotzsch A., Schliermann A. (2015). GDF-5 can act as a context-dependent BMP-2 antagonist. BMC Biol.

[77] Magro G. (2020). SARS-CoV-2 and COVID-19: Is interleukin-6 (IL-6) the ‘culprit lesion’ of ARDS onset? What is there besides tocilizumab? SGP130Fc. Cytokine X.

[78] Chen L.Y.C., Hoiland R.L., Stukas S., Wellington C.L., Sekhon M.S. (2020). Confronting the controversy: interleukin-6 and the COVID-19 cytokine storm syndrome. Eur Respir J.

[79] Zhang X., Wu K., Wang D., Yue X., Song D., Zhu Y. (2007). Nucleocapsid protein of SARS-CoV activates interleukin-6 expression through cellular transcription factor NF-kappaB. Virology.

[80] Mendoza J.L., Schneider W.M., Hoffmann H.-H., Vercauteren K., Jude K.M., Xiong A. (2017). The IFN-λ-IFN-λR1-IL-10Rβ complex reveals structural features underlying type III IFN functional plasticity. Immunity.

[81] Corbett A.J., Eckle S.B.G., Birkinshaw R.W., Liu L., Patel O., Mahony J. (2014). T-cell activation by transitory neo-antigens derived from distinct microbial pathways. Nature.

[82] Yu G., Boone T., Delaney J., Hawkins N., Kelley M., Ramakrishnan M. (2000). APRIL and TALL-I and receptors BCMA and TACI: system for regulating humoral immunity. Nat Immunol.

[83] Tamada K., Shimozaki K., Chapoval A.I., Zhai Y., Su J., Chen S.-F. (2000). LIGHT, a TNF-like molecule, costimulates T cell proliferation and is required for dendritic cell-mediated allogeneic T cell response. J Immunol.

[84] Smart O.S., Womack T.O., Flensburg C., Keller P., Paciorek W., Sharff A. (2012). Exploiting structure similarity in refinement: automated NCS and target-structure restraints in {\it BUSTER}. Acta Crystallogr Sect D.

[85] Nonaka M., Yoshizaki F. (2004). Evolution of the complement system. Mol Immunol.

[86] Bernet J., Mullick J., Singh A.K., Sahu A. (2003). Viral mimicry of the complement system. J Biosci.

[87] Java A., Apicelli A.J., Kathryn Liszewski M., Coler-Reilly A., Atkinson J.P., Kim A.H.J. (2020). The complement system in COVID-19: Friend and foe?. JCI Insight.

[88] Magro C., Mulvey J.J., Berlin D., Nuovo G., Salvatore S., Harp J. (2020). Complement associated microvascular injury and thrombosis in the pathogenesis of severe COVID-19 infection: a report of five cases. Transl Res.

[89] Ramlall V., Thangaraj P.M., Meydan C., Foox J., Butler D., May B. (2020). Identification of Immune complement function as a determinant of adverse SARS-CoV-2 infection outcome. MedRxiv.

[90] Yu J., Yuan X., Chen H., Chaturvedi S., Braunstein E.M., Brodsky R.A. (2020). Direct activation of the alternative complement pathway by SARS-CoV-2 spike proteins is blocked by factor D inhibition. Blood.

[91] Xue X., Wu J., Ricklin D., Forneris F., Di Crescenzio P., Schmidt C.Q. (2017). Regulator-dependent mechanisms of C3b processing by factor I allow differentiation of immune responses. Nat Struct Mol Biol.

[92] van den Elsen J.M.H., Isenman D.E. (2011). A crystal structure of the complex between human complement receptor 2 and its ligand C3d. Science (80-).

[93] Campbell I.D., Spitzfaden C. (1994). Building proteins with fibronectin type III modules. Structure.

[94] Leahy D.J., Aukhil I., Erickson H.P. (1996). Crystal structure of a four-domain segment of human fibronectin encompassing the RGD loop and synergy region. Cell.

[95] Mendiratta S.S., Sekulic N., Hernandez-Guzman F.G., Close B.E., Lavie A., Colley K.J. (2006). A Novel α-helix in the first fibronectin type III repeat of the neural cell adhesion molecule is critical for N-glycan polysialylation. J Biol Chem.

[96] Luca V.C., Kim B.C., Ge C., Kakuda S., Wu D.i., Roein-Peikar M. (2017). Notch-Jagged complex structure implicates a catch bond in tuning ligand sensitivity. Science.

[97] Renko M., Sabotič J., Mihelič M., Brzin J., Kos J., Turk D. (2010). Versatile loops in mycocypins inhibit three protease families. J Biol Chem.

[98] Pišlar A., Mitrović A., Sabotič J., Pečar Fonović U., Perišić Nanut M., Jakoš T. (2020). The role of cysteine peptidases in coronavirus cell entry and replication: the therapeutic potential of cathepsin inhibitors. PLOS Pathog.

[99] Cheng Y., Zak O., Aisen P., Harrison S.C., Walz T. (2004). Structure of the human transferrin receptor-transferrin complex. Cell.

[100] Watson A.A., Lebedev A.A., Hall B.A., Fenton-May A.E., Vagin A.A., Dejnirattisai W. (2011). Structural flexibility of the macrophage dengue virus receptor CLEC5A: implications for ligand binding and signaling*. J Biol Chem.

[101] Zhang H., Casasnovas J.M., Jin M., Liu J.-H., Gahmberg C.G., Springer T.A. (2008). An unusual allosteric mobility of the c-terminal helix of a high-affinity αl integrin i domain variant bound to ICAM-5. Mol Cell.

[102] Changeux J.-P., Amoura Z., Rey F.A., Miyara M. (2020). A nicotinic hypothesis for Covid-19 with preventive and therapeutic implications. C R Biol.

[103] Pizarro J.C., Chitarra V., Verger D., Holm I., Pêtres S., Dartevelle S. (2003). Crystal structure of a fab complex formed with PfMSP1-19, the C-terminal fragment of merozoite surface protein 1 from plasmodium falciparum: a malaria vaccine candidate. J Mol Biol.

[104] Igonet S., Vulliez-Le Normand B., Faure G., Riottot M.-M., Kocken C.H.M., Thomas A.W. (2007). Cross-reactivity studies of an anti-plasmodium vivax apical membrane antigen 1 monoclonal antibody: binding and structural characterisation. J Mol Biol.

[105] Craig L., Sanschagrin P.C., Rozek A., Lackie S., Kuhn L.A., Scott J.K. (1998). The role of structure in antibody cross-reactivity between peptides and folded proteins. J Mol Biol.

[106] Panda A.K., Tripathy R., Das B.K. (2020). Plasmodium falciparum infection may protect a population from severe acute respiratory syndrome coronavirus 2 infection. J Infect Dis.

[107] Iesa M.A.M., Osman M.E.M., Hassan M.A., Dirar A.I.A., Abuzeid N., Mancuso J.J. (2020). SARS-CoV-2 and plasmodium falciparum common immunodominant regions may explain low COVID-19 incidence in the malaria-endemic belt. New Microbes New Infect.

[108] Kalungi A., Kinyanda E., Akena D.H., Kaleebu P., Bisangwa I.M. (2021). Less severe cases of COVID-19 in sub-saharan africa: could co-infection or a recent history of plasmodium falciparum infection be protective?. Front Immunol.

[109] Raham T.F. (2021). Influence of malaria endemicity and tuberculosis prevalence on COVID-19 mortality. Public Health.

[110] Jankowiak Ł., Rozsa L., Tryjanowski P., Møller A.P. (2020). A negative covariation between toxoplasmosis and CoVID-19 with alternative interpretations. Sci Rep.

[111] Ploss A., Evans M.J. (2012). Hepatitis C virus host cell entry. Curr Opin Virol.

[112] Heile JM, Fong Y-L, Rosa D, Berger K, Saletti G, Campagnoli S, et al. Evaluation of Hepatitis C Virus Glycoprotein E2 for Vaccine Design: an Endoplasmic Reticulum-Retained Recombinant Protein Is Superior to Secreted Recombinant Protein and DNA-Based Vaccine Candidates. J Virol 2000;74:6885 LP – 6892. https://doi.org/10.1128/JVI.74.15.6885-6892.2000. 10.1128/jvi.74.15.6885-6892.2000 PMC112206 10888628

[113] Richardson S., Hirsch J.S., Narasimhan M., Crawford J.M., McGinn T., Davidson K.W. (2020). Presenting characteristics, comorbidities, and outcomes among 5700 patients hospitalized with COVID-19 in the New York City Area. JAMA.

[114] Reddy K.R. (2020). SARS-CoV-2 and the liver: considerations in hepatitis b and hepatitis C infections. Clin Liver Dis.

[115] Mirzaie H, Vahidi M, Shokoohi M, Darvishian M, Sharifi H, Sharafi H, et al. COVID-19 among patients with hepatitis B or hepatitis C: A systematic review. MedRxiv 2020:2020.10.22.20216317. https://doi.org/10.1101/2020.10.22.20216317.

[116] Brockbank SM V, Soden J, Faba-Rodriguez R, Ribeiro LR, Geh C, Thomas H, et al. SARS-CoV-2 comprehensive receptor profiling: mechanistic insight to drive new therapeutic strategies. BioRxiv 2021:2021.03.11.434937. https://doi.org/10.1101/2021.03.11.434937.

[117] Abraham J., Corbett K.D., Farzan M., Choe H., Harrison S.C. (2010). Structural basis for receptor recognition by New World hemorrhagic fever arenaviruses. Nat Struct Mol Biol.

[118] Moonens K., Gideonsson P., Subedi S., Bugaytsova J., Romaõ E., Mendez M. (2016). Structural insights into polymorphic ABO glycan binding by helicobacter pylori. Cell Host Microbe.

[119] Saez N.J., Mobli M., Bieri M., Chassagnon I.R., Malde A.K., Gamsjaeger R. (2011). A dynamic pharmacophore drives the interaction between Psalmotoxin-1 and the putative drug target acid-sensing ion channel 1a. Mol Pharmacol.

[120] Volpon L., Lamthanh H., Barbier J., Gilles N., Molgó J., Ménez A. (2004). NMR solution structures of δ-conotoxin EVIA from conus ermineus that selectively acts on vertebrate neuronal Na+ channels. J Biol Chem.

[121] Dellisanti C.D., Yao Y., Stroud J.C., Wang Z.-Z., Chen L. (2007). Crystal structure of the extracellular domain of nAChR alpha1 bound to alpha-bungarotoxin at 1.94 A resolution. Nat Neurosci.

[122] Lee S.-C., Lin C.-C., Wang C.-H., Wu P.-L., Huang H.-W., Chang C.-I. (2014). Endocytotic routes of cobra cardiotoxins depend on spatial distribution of positively charged and hydrophobic domains to target distinct types of sulfated glycoconjugates on cell surface. J Biol Chem.

[123] Corfield P.W., Lee T.J., Low B.W. (1989). The crystal structure of erabutoxin a at 2.0-A resolution. J Biol Chem.

[124] McFarland A.J., Yousuf M.S., Shiers S., Price T.J. (2021). Neurobiology of SARS-CoV-2 interactions with the peripheral nervous system: implications for COVID-19 and pain. PAIN Rep.

[125] Moutal A., Martin L.F., Boinon L., Gomez K., Ran D., Zhou Y. (2021). SARS-CoV-2 Spike protein co-opts VEGF-A/Neuropilin-1 receptor signaling to induce analgesia. Pain.

[126] Iba T., Levy J.H., Levi M., Connors J.M., Thachil J. (2020). Coagulopathy of coronavirus disease 2019. Crit Care Med.

[127] Parvizpour S., Pourseif M.M., Razmara J., Rafi M.A., Omidi Y. (2020). Epitope-based vaccine design: a comprehensive overview of bioinformatics approaches. Drug Discov Today.

[128] Mastellos D., Morikis D., Isaacs S.N., Holland M.C., Strey C.W., Lambris J.D. (2003). Complement: structure, functions, evolution, and viral molecular mimicry. Immunol Res.

[129] Rowan E.G. (2001). What does beta-bungarotoxin do at the neuromuscular junction?. Toxicon.

[130] Dongol Y., Caldas Cardoso F., Lewis R.J. (2019). Spider knottin pharmacology at voltage-gated sodium channels and their potential to modulate pain pathways. Toxins (Basel).

[131] Wu P.-L., Lee S.-C., Chuang C.-C., Mori S., Akakura N., Wu W. (2006). Non-cytotoxic cobra cardiotoxin A5 binds to alpha(v)beta3 integrin and inhibits bone resorption. Identification of cardiotoxins as non-RGD integrin-binding proteins of the Ly-6 family. J Biol Chem.

[132] Cao L, Goreshnik I, Coventry B, Case JB, Miller L, Kozodoy L, et al. De novo design of picomolar SARS-CoV-2 miniprotein inhibitors. Science (80-) 2020;370:426 LP – 431. https://doi.org/10.1126/science.abd9909. 10.1126/science.abd9909 PMC7857403 32907861

[133] Brister J.R., Ako-Adjei D., Bao Y., Blinkova O. (2015). NCBI viral genomes resource. Nucleic Acids Res.

[134] Torres P.H.M., Rossi A.D., Blundell T.L. (2021). ProtCHOIR: a tool for proteome-scale generation of homo-oligomers. Brief Bioinform.

[135] Šali A., Blundell T.L. (1993). Comparative protein modelling by satisfaction of spatial restraints. J Mol Biol.

[136] Krissinel E., Henrick K. (2007). Inference of macromolecular assemblies from crystalline state. J Mol Biol.

[137] Altschul S.F., Madden T.L., Schäffer A.A., Zhang J., Zhang Z., Miller W. (1997). Gapped BLAST and PSI-BLAST: a new generation of protein database search programs. Nucleic Acids Res.

[138] Krissinel E. (2012). Enhanced fold recognition using efficient short fragment clustering. J Mol Biochem.

[139] Chen V.B., Arendall W.B., Headd J.J., Keedy D.A., Immormino R.M., Kapral G.J. (2010). MolProbity: all-atom structure validation for macromolecular crystallography. Acta Crystallogr Sect D Biol Crystallogr.

[140] Šali A. MODELLER: A Program for Protein Structure Modeling 2019:779–815.

[141] Madeira F, Park YM, Lee J, Buso N, Gur T, Madhusoodanan N, et al. The EMBL-EBI search and sequence analysis tools APIs in 2019. Nucleic Acids Res 2019;47:W636–41. https://doi.org/10.1093/nar/gkz268. 10.1093/nar/gkz268 PMC6602479 30976793

[142] Alsulami AF, Thomas SE, Jamasb AR, Beaudoin CA, Moghul I, Bannerman B, et al. SARS-CoV-2 3D database: understanding the coronavirus proteome and evaluating possible drug targets. Brief Bioinform 2021;22:769–80. https://doi.org/10.1093/bib/bbaa404. 10.1093/bib/bbaa404 PMC7929435 33416848

[143] Steentoft C., Vakhrushev S.Y., Joshi H.J., Kong Y., Vester-Christensen M.B., Schjoldager K.-B G. (2013). Precision mapping of the human O-GalNAc glycoproteome through SimpleCell technology. EMBO J.

[144] Gupta R, Jung E, Brunak S. Prediction of N-glycosylation sites in human proteins. In preparation. 2004.

[145] Kuriata A, Gierut AM, Oleniecki T, Ciemny MP, Kolinski A, Kurcinski M, et al. CABS-flex 2.0: a web server for fast simulations of flexibility of protein structures. Nucleic Acids Res 2018;46:W338–43. https://doi.org/10.1093/nar/gky356. 10.1093/nar/gky356 PMC6031000 29762700

[146] Kmiecik S., Gront D., Kolinski M., Wieteska L., Dawid A.E., Kolinski A. (2016). Coarse-grained protein models and their applications. Chem Rev.

[147] Holm L., Laakso L.M. (2016). Dali server update. Nucleic Acids Res.

[148] Li Z., Jaroszewski L., Iyer M., Sedova M., Godzik A. (2020). FATCAT 2.0: towards a better understanding of the structural diversity of proteins. Nucleic Acids Res.

[149] Lo W.-C., Lee C.-Y., Lee C.-C., Lyu P.-C. (2009). iSARST: an integrated SARST web server for rapid protein structural similarity searches. Nucleic Acids Res.

[150] Deng L., Zhong G., Liu C., Luo J., Liu H. (2019). MADOKA: An ultra-fast approach for large-scale protein structure similarity searching. BMC Bioinf.

[151] Krissinel E., Henrick K. (2004). Secondary-structure matching (SSM), a new tool for fast protein structure alignment in three dimensions. Acta Crystallogr Sect D Biol Crystallogr.

[152] Zhang Y., Skolnick J. (2005). TM-align: a protein structure alignment algorithm based on the TM-score. Nucleic Acids Res.

[153] Ayoub R., Lee Y., Zhang Y. (2019). Rupee: A fast and accurate purely geometric protein structure search. PLoS ONE.

[154] Dong R., Pan S., Peng Z., Zhang Y., Yang J. (2018). MTM-align: a server for fast protein structure database search and multiple protein structure alignment. Nucleic Acids Res.

[155] Kabsch W., Sander C. (1983). Dictionary of protein secondary structure: Pattern recognition of hydrogen-bonded and geometrical features. Biopolymers.

[156] Zhang Y., Skolnick J. (2004). Scoring function for automated assessment of protein structure template quality. Proteins.

[157] Hutchinson E.G., Thornton J.M. (1996). PROMOTIF–a program to identify and analyze structural motifs in proteins. Protein Sci.

[158] Krissinel E, Henrick K. Multiple Alignment of Protein Structures in Three Dimensions BT - Computational Life Sciences. In: R. Berthold M, Glen RC, Diederichs K, Kohlbacher O, Fischer I, editors., Berlin, Heidelberg: Springer Berlin Heidelberg; 2005, p. 67–78.

[159] Sillitoe I, Dawson N, Lewis TE, Das S, Lees JG, Ashford P, et al. CATH: Expanding the horizons of structure-based functional annotations for genome sequences. Nucleic Acids Res 2019;47:D280–4. https://doi.org/10.1093/nar/gky1097. 10.1093/nar/gky1097 PMC6323983 30398663

[160] Andreeva A., Kulesha E., Gough J., Murzin A.G. (2020). The SCOP database in 2020: expanded classification of representative family and superfamily domains of known protein structures. Nucleic Acids Res.

[161] Cheng H., Schaeffer R.D., Liao Y., Kinch L.N., Pei J., Shi S. (2014). ECOD: an evolutionary classification of protein domains. PLoS Comput Biol.

[162] Burley SK, Berman HM, Bhikadiya C, Bi C, Chen L, Di Costanzo L, et al. RCSB Protein Data Bank: Biological macromolecular structures enabling research and education in fundamental biology, biomedicine, biotechnology and energy. Nucleic Acids Res 2019;47:D464–74. https://doi.org/10.1093/nar/gky1004. 10.1093/nar/gky1004 PMC6324064 30357411

[163] Guven-Maiorov E., Hakouz A., Valjevac S., Keskin O., Tsai C.-J., Gursoy A. (2020). HMI-PRED: a web server for structural prediction of host-microbe interactions based on interface mimicry. J Mol Biol.

[164] Hubbard S, Thornton J. NACCESS, Department of Biochemistry Molecular Biology, University College London 1993.

[165] Wang C., Bradley P., Baker D. (2007). Protein-protein docking with backbone flexibility. J Mol Biol.

[166] Bateman A. (2019). UniProt: a worldwide hub of protein knowledge. Nucleic Acids Res.

[167] Kozakov D., Hallc D.R., Xiab B., Porterb K.A., Padhornya D., Yuehb C. (2017). The ClusPro web server for protein-protein docking. Nat Protoc.

[168] Vajda S., Yueh C., Beglov D., Bohnuud T., Mottarella S.E., Xia B. (2017). New additions to the ClusPro server motivated by CAPRI. Proteins Struct Funct Bioinforma.

[169] Desta I.T., Porter K.A., Xia B., Kozakov D., Vajda S. (2020). Performance and Its Limits in Rigid Body Protein-Protein Docking. Structure.

[170] Kozakov D., Beglov D., Bohnuud T., Mottarella S.E., Xia B., Hall D.R. (2013). How good is automated protein docking?. Proteins.

[171] Szklarczyk D, Gable AL, Lyon D, Junge A, Wyder S, Huerta-Cepas J, et al. STRING v11: protein–protein association networks with increased coverage, supporting functional discovery in genome-wide experimental datasets. Nucleic Acids Res 2019;47:D607–13. https://doi.org/10.1093/nar/gky1131. 10.1093/nar/gky1131 PMC6323986 30476243

[172] Schymkowitz J., Borg J., Stricher F., Nys R., Rousseau F., Serrano L. (2005). The FoldX web server: an online force field. Nucleic Acids Res.

[173] Stout T. Show charged amino acids on PyMOL 2004.

[174] Rodrigues J, Teixeira JMC, Trellet M, Bonvin A. pdb-tools: a swiss army knife for molecular structures. F1000Research 2018;7. https://doi.org/10.12688/f1000research.17456.1. 10.12688/f1000research.17456.1 PMC6343223 30705752

