# Supplementary material for: Predicted structural mimicry of spike receptor-binding motifs from highly pathogenic human coronaviruses
Source: Comput Struct Biotechnol J. 2021 Jul 2;19:3938–53. doi: 10.1016/j.csbj.2021.06.041 (PMC8249111; doi:10.1016/j.csbj.2021.06.041)
Supplement: Supplementary data 1 [file mmc1.docx]

**Supplementary figures and tables**


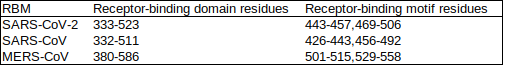


Supplementary Table 1. Spike protein receptor-binding domain and motif residues selected in this study


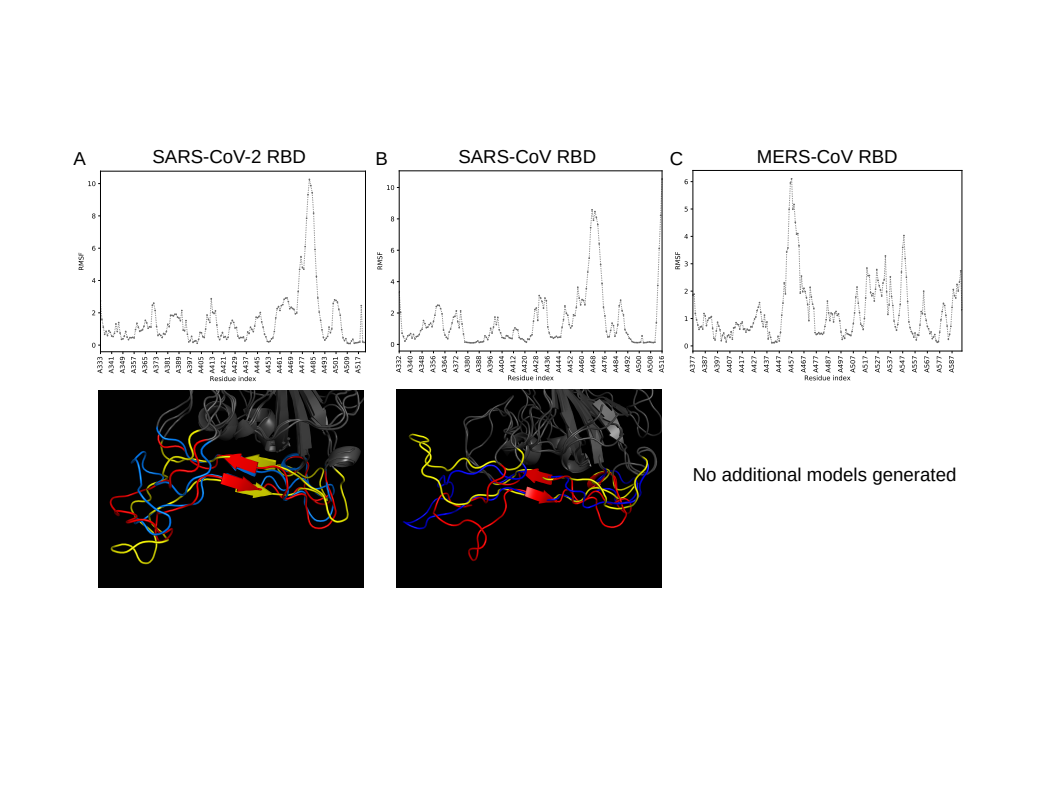


Supplementary Figure 1. CABS-Flex 2.0 analysis and models for coronavirus RBDs

The top row displays the root mean square flexibility for each residue in the SARS-CoV-2 (A), SARS-CoV (B), and MERS-CoV (C) RBDs in the CABS-Flex 2.0 analyses. The bottom row shows selected models from CABS-Flex 2.0, included for the SARS-CoV-2 and SARS-CoV structural similarity screen, in alignment with the reference RBM.


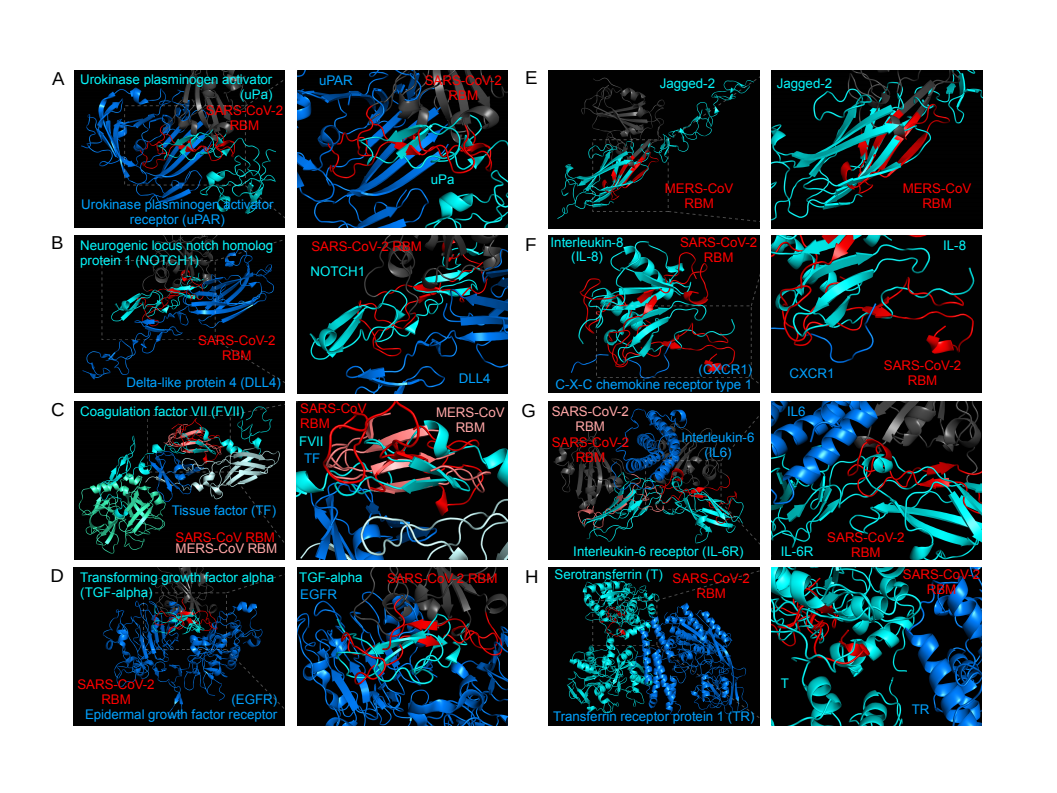
Supplementary Figure 2. Structural alignments of potential endogenous mimics

Structural alignments between the following coronavirus RBMs and potential molecular mimics are shown: SARS-CoV-2 and urokinase plasminogen activator (A), SARS-CoV-2 and NOTCH1 (B), MERS-CoV and SARS-CoV mimicking coagulation factor VIIa (C), SARS-CoV-2 and TGF-alpha (D), MERS-CoV and jagged-2 (E), SARS-CoV-2 and IL-8 (F), SARS-CoV-2 and IL-6 receptor alpha and beta chains (G), SARS-CoV-2 and serotransferrin (H). RBMs are labelled red, mimicked proteins are cyan, and potential interaction partners are marine blue.


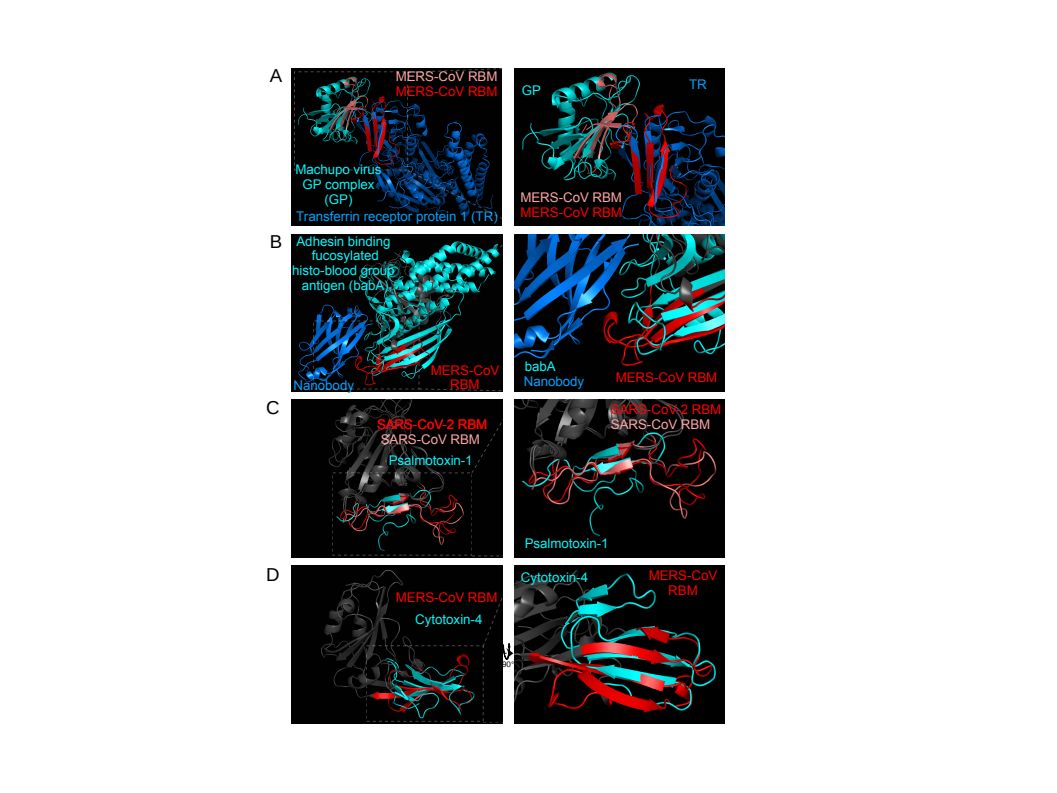


Supplementary Figure 3. Structural alignments with exogenous proteins

The MERS-CoV RBM aligned to the transferrin receptor protein 1 and Machupo virus glycoprotein polyprotein GP complex (A) and the adhesin-binding fucosylated histo-blood group antigen (B). The SARS-CoV and SARS-CoV-2 RBMs aligned to psalmotoxin-1 (C), and the MERS-CoV RBM aligned to cytotoxin 4 (D). RBMs are labelled red, mimicked proteins are cyan, and potential interaction partners are marine blue.


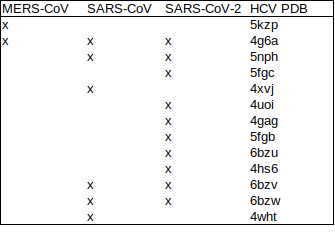


Supplementary Table 2. Hepatitis C virus (HCV) PDB codes returned in structural similarity screen
